# Genotyping, generation and proteomic profiling of the first human autosomal dominant osteopetrosis type II-specific induced pluripotent stem cells

**DOI:** 10.1186/s13287-019-1369-8

**Published:** 2019-08-14

**Authors:** Minglin Ou, Chunhong Li, Donge Tang, Wen Xue, Yong Xu, Peng Zhu, Bo Li, Jiansheng Xie, Jiejing Chen, Weiguo Sui, Lianghong Yin, Yong Dai

**Affiliations:** 1 Clinical Medical Research Center of The Second Clinical Medical College, Jinan University, Shenzhen People’s Hospital, No.1017, Dongmen North Road, Luohu District, Shenzhen, 518020 China; 2 Guangxi Key Laboratory of Metabolic Disease Research, Central Laboratory of Guilin No. 181 Hospital, No. 1, Xinqiaoyuan Road, Guilin, 541002 China; 3 Department of Nephrology, The First Affiliated Hospital of Jinan University, Guangzhou, 510630 China; 4 Shenzen Maternity & Child Healthcare Hospital, Shenzhen, 518017 China; 5 College of Life Science, Guangxi Normal University, Guilin, 541004 China

**Keywords:** Osteopetrosis, Whole-exome sequencing, CLCN7, iPSCs, Proteomics, 2-hydroxyisobutyrylation

## Abstract

**Background:**

Autosomal dominant osteopetrosis type II (ADO2) is a rare human genetic disease that has been broadly studied as an important osteopetrosis model; however, there are no disease-specific induced pluripotent stem cells (ADO2-iPSCs) that may be valuable for understanding the pathogenesis and may be a potential source of cells for autologous cell-based therapies.

**Methods:**

To generate the first human ADO2-iPSCs from a Chinese family with ADO2 and to identify their characteristics, blood samples were collected from the proband and his parents and were used for genotyping by whole-exome sequencing (WES); the urine-derived cells of the proband were reprogrammed with episomal plasmids that contained transcription factors, such as KLF4, OCT4, c-MYC, and SOX2. The proteome-wide protein quantification and lysine 2-hydroxyisobutyrylation detection of the ADO2-iPSCs and normal control iPSCs (NC-iPSCs) were performed by high-resolution LC-MS/MS and bioinformatics analysis.

**Results:**

WES with filtering strategies identified a mutation in CLCN7 (R286W) in the proband and his father, which was absent in the proband’s mother and the healthy controls; this was confirmed by Sanger sequencing. The ADO2-iPSCs were successfully generated, which carried a normal male karyotype (46, XY) and the mutation of CLCN7 (R286W); the ADO2-iPSCs positively expressed alkaline phosphatase and other surface markers; and no vector and transgene were detected. The ADO2-iPSCs could differentiate into all three germ cell layers, both in vitro and in vivo. The proteomic profiling revealed similar expression of pluripotency markers in the two cell lines and identified 7405 proteins and 3664 2-hydroxyisobutyrylated peptides in 1036 proteins in the ADO2-iPSCs.

**Conclusions:**

Our data indicated that the mutation CLCN7 (R286W) may be a cause of the osteopetrosis family. The generated vector-free and transgene-free ADO2-iPSCs with known proteomic characteristics may be valuable for personalized and cell-based regenerative medicine in the future.

**Electronic supplementary material:**

The online version of this article (10.1186/s13287-019-1369-8) contains supplementary material, which is available to authorized users.

## Background

Osteopetrosis is a group of rare human genetic diseases that are characterized by abnormal bone density on radiographs [1]. It is also a heterogeneous disease, and patients with osteopetrosis may present with different forms of severity that range from asymptomatic to fatal [2]. It is difficult to understand the exact pathologic process of osteopetrosis because this is a rare disease, and the generation of animal models may be technically challenging and may fail to completely replicate the clinical features. In the clinic, the patients with more severe conditions were commonly observed as autosomal recessive osteopetrosis (ARO), and those with mild conditions were more commonly found in adults with autosomal dominant osteopetrosis type II (ADO2) [3]. Presently, allogeneic hematopoietic stem cell transplantation (HSCT) treatments have been chosen for the treatment of severe osteopetrosis, which results in 73% of patients achieving 5 years of disease-free survival [4]. This kind of treatment has been greatly improved over the past few years, but the engraftment of mesenchymal stem cells from donors may have unexpected difficulties, allogeneic HSCT is still a dangerous procedure with other kinds of toxicities and is limited by the requirement of a matched donor [5, 6]. Therefore, these may be some of the main reasons why, until now, there were no studies that focused on determining how HSCT works in the severe cases of ADO2. In theory, ADO2 may be treated by autologous induced pluripotent stem cell (iPSC)-based cell therapies as a hematologic disorder [7]. Recently, experimental evidences have revealed that autologous induced pluripotent stem cells (iPSCs) can be generated from somatic cells with origins from the mesoderm, ectoderm, and endoderm, including human urine-derived cells [8]. It is important that the urine can be obtained by a noninvasive procedure, and patient urinary iPSCs have been found valuably in disease modeling and regenerative medicine [9].

However, the disease-specific urinary iPSCs should be well characterized before they could be used for studies or other applications. Recently, some studies have indicated that quantitative proteomic analysis of iPSCs were valuable in cell characterizing systematically and discovering potential molecular mechanism associated with pathology, because affecting cellular processes in human disease have been found in undifferentiated iPSCs generated from patient’s somatic cells [10, 11]. In practice, mass spectrometry (MS)-based proteomics have been developed and enabled to the study the panoramic views of protein expression and modifications, including the 2-hydroxyisobutyrylation (K_hib_), which is conserved proteome-wide and may be one of the most important post-translational modifications (PTMs) [12]. Therefore, proteomic profiling involving protein identification and K_hib_ detection may be a benefit for us to study the cellular biology of human disease-specific iPSCs.

Here, we performed genotyping of an osteopetrosis family by whole-exome sequencing (WES) and tried to generate disease-specific iPSCs using urine-derived cells from one ADO2 family; we analyzed their characteristics, including the global proteome using LC-MS/MS analysis, which may be valuable for understanding the autosomal dominant osteopetrosis type II specific induced pluripotent stem cell (ADO2-iPSC) biology characteristics and therapy of ADO2 in the future.

## Materials and methods

### Human samples

**Fig. 1:**
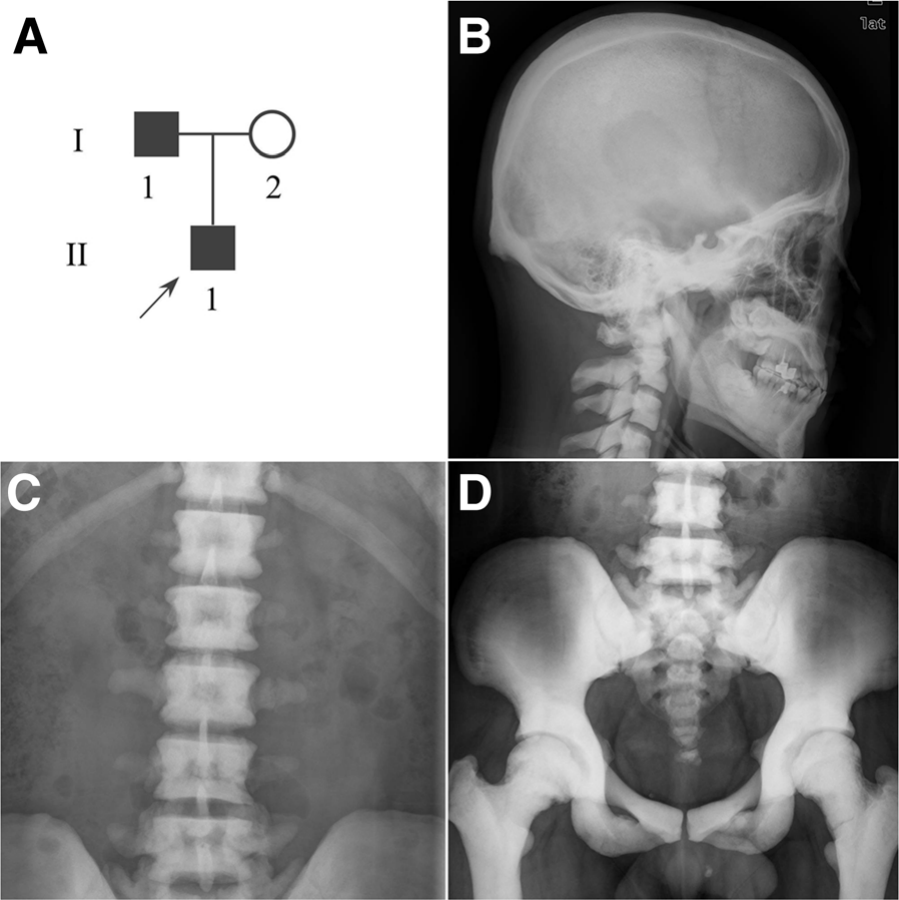
The pedigree and the radiological features of the proband. **a** The arrow in the pedigree indicates the proband. **b** Diffuse and dense sclerosis of the skull. **c** The lumbar spine with the appearance of classic vertebral endplate thickening. **d** The marked sclerosis at acetabulum and iliac wings

Informed consent was obtained from the participant donors in a family with ADO2 (Fig. 1), including the proband (II1, a 31-year-old male), and his parents. The diagnosis of ADO2 was confirmed by standard spine and pelvis radiographs and genotyping [1]. The proband was obviously affected by general skeletal sclerosis and his father had mild clinical features. The venous blood samples were taken from donors for the purpose of genetic diagnosis, and the fresh urine cells were collected from the proband for reprogramming after genetic diagnosis. For the urine cell collection, the urethral area of the ADO2 patient was washed, and the middle stream of the random urine samples of the day was collected using a sterile container; the required volume of the sample was at least 200 mL. Genomic DNA was extracted with a QIAamp DNA Mini Kit (Qiagen, Hilden, Germany) using standard procedures.

### Genotyping by WES

Exome capture of samples from the proband and his parents was performed as previously described in our previous studies with minor modifications [1, 13]. Briefly, the extracted DNA samples were randomly fragmented with the size of fragments between 150 and 250 bp; the “A” base was added to the 3′-end of each strand for DNA fragment repair; ligation-mediated PCR (LM-PCR) was performed after adapter ligation and size selection; then the product of LM-PCR was purified and hybridized to the array for the enrichment of the exome; the captured DNA fragments were then circularized, and rolling circle amplification (RCA) was performed for the generation of DNA nanoballs. Each qualified, captured library was subjected to high-throughput sequencing using BGISEQ-500 platforms (BGI, Wuhan, China). The raw data were produced and processed by BGISEQ-500 basecalling software and were stored in the FASTQ format. Quality control was performed for the whole pipeline, the raw data were filtered, and the clean data were mapped to the human reference genome (GRCh37/HG19) by Burrows-Wheeler Aligner (BWA) software (V0.7.15) [14, 15]. To ensure the accuracy of variant calling, the recommended variant analysis of Genome Analysis Toolkit (v3.3.0) (GATK; https://www.broadinstitute.org/gatk/guide/best-practices) was used; GATK was also used for local realignment including base quality score recalibration and InDels [16, 17]. The duplicate reads were excluded by Picard Tools (http://broadinstitute.github.io/picard/). The coverage and depth of sequencing of each sample were calculated based on the data from the alignments. The SnpEff tool (http://snpeff.sourceforge.net/SnpEff\cr_manual.html) was used for variant annotations, and the final variants and the annotation results were used for downstream advanced analysis. The discovered SNPs and InDels were compared to those in the NCBI dbSNP (v141), 1000 Genomes Project, and NHLBI Grand Opportunity Exome Sequencing Project 6500 (ESP6500) databases and were further filtered by minor allele frequency (MAF). The candidate mutations were identified by determining which variants were present in the ADO2 patients and which were absent in the healthy controls based on the list of known osteopetrotic genes.

### CLCN7 mutation confirmation

The candidate mutation of ADO2 in the genome of the proband and his family members was confirmed by PCR and Sanger sequencing as described in our previous study [1]. Briefly, the PCR primers of CLCN7 were designed to amplify the DNA sequence with the candidate mutation CLCN7 (R286W) (Table 1). The buffer was mixed with DNA, a dNTP mixture, Taq polymerase, and MgCl_2_ and was amplified by a thermal cycler, MyCycler (Bio-Rad, Hercules, CA, USA), with the standard conditions, and was then analyzed by an ABI Prism 3730 DNA Analyzer (Applied Biosystems, Foster City, CA, USA) with the standard procedures.

**Table 1:** The primers used for PCR amplification

No.	Target	Sequence	Product sizes (bp)
1	CLCN7	F: ACCCAGACCACGTCAGAAAG	411
		R: GACTCGGTTGTCCTGAAAGC	
2	SeV	F: GGA TCA CTA GGT GAT ATC GAG C	181
		R: ACC AGA CAA GAG TTT AAG AGA TAT GTA TC	
3	KOS	F: ATG CAC CGC TAC GAC GTG AGC GC	528
		R: ACC TTG ACA ATC CTG ATG TGG	
4	Klf4	F: TTC CTG CAT GCC AGA GGA GCC C	410
		R: AAT GTA TCG AAG GTG CTC AA	
5	c-Myc	F: TAA CTG ACT AGC AGG CTT GTC G	532
		R: TCC ACA TAC AGT CCT GGA TGA TGA TG	
6	OCT4	F: CCTCACTTCACTGCACTGTA	164
		R: CCTCACTTCACTGCACTGTA	
7	GATA4	F: GACAATCTGGTTAGGGGAAGC	105
		R: GAGAGATGCAGTGTGCTCGT	
8	MSX1	F: TGCCTCGCTCTACGGTGCCT	154
		R: GGCTGGAGGAATCGGCTGGC	
9	SOX1	F: TTTCCCCTCGCTTTCTCA	104
		R: TGCAGGCTGAATTCGGTT	
10	GAPDH	F: GGAGCGAGATCCCTCCAAAAT	197
		R: GGCTGTTGTCATACTTCTCATGG	

Note: *F* forward primer, *R* reverse primer

### Urine cell culture and generation of ADO2-iPSCs

The urine sample was dispensed into 50-mL tubes and was centrifuged for 10 min at room temperature at 300×*g*. The supernatant was discarded carefully and approximately 5 mL of the sample was kept in the tube. The supernatant (with the remaining cells) was resuspended, transferred, and pooled into one 50-mL tube and was centrifuged again for 10 min (300×*g*). The supernatant was carefully discarded; the cells in the bottom of the tube were washed and resuspended using 0.5 mL Urineasy Medium (Cellapy, Beijing, China) and were seeded onto culture plates (35 mm). They were cultured with 5% CO_2_ (37 °C); approximately 2 mL of medium was added at the beginning of the first 24 h of culture, and the medium was carefully changed every 60 h. The cultured cells were then seeded into a 6-well plate, and they were reprogrammed when they were grown to 50–80% confluence. The generation of ADO2-specific iPSCs was performed by infecting the cells with nonintegrating Sendai virus (SeV)-mediated transfection (CytoTune2.0 Sendai vectors; Thermo Scientific), which contained the 4 canonical transcriptional factors such as OCT4, SOX2, KLF4, and c-MYC according to the manufacturer’s instructions. Briefly, approximately 3 × 10^5^ urine cells were infected at a multiplicity of infection of 5 and were incubated for 24 h. The cells were collected on the following day, seeded in fresh medium (day 1), and cultured for 6 days (fed by fresh medium every 2 days); 7 days posttransduction (day 7), the cells were collected and seeded onto plates coated with Matrigel in Urineasy Medium (Cellapy, Beijing, China) for 24 h at 37 °C with 5% CO_2_. Then, the cells (day 8) were grown in Reproeasy culture medium with growth factors (Cellapy, China). The ADO2-iPSC colonies were manually selected based on their morphology between day 14 and day 28 postinfection and were maintained in the culture medium. In our present study, three different clones were picked on day 17 after plasmid infection (passage number = 0, P0), and the best one among the three clones in the latter passage (passage number = 10, P10) was used to establish the ADO2-iPS cell line.

### Short tandem repeat profiling

To confirm the origin of the new iPSC line, the extracted DNAs from the blood of the proband (ADO2-Blood) and from the ADO2-iPSCs were used to perform short tandem repeat (STR) profiling. The genetic signatures were analyzed using the PowerPlex® 21 PCR Amplification System (Promega) based on the 21 loci markers. The PCR products were tested by an ABI 3500 genetic analyzer (Applied Biosystems, Life Technologies), and the output data were analyzed by GeneMapper® ID Software (Applied Biosystems, Life Technologies) according to the manufacturer’s instructions.

### Cell staining and immunofluorescence

The alkaline phosphatase staining was performed using a BCIP/NBT Alkaline Phosphatase Color Development Kit (Leagene, Beijing, China). For immunofluorescence, the cells that were cultured in human PSCeasy Medium (Cellapy, Beijing, China) were harvested and fixed with phosphate-buffered saline (PBS) and paraformaldehyde (4%) for 15 min at room temperature. For the molecules localized in the nucleus, the cells were treated with Triton X-100 (0.5%) for 15 min and with BSA (3%) for 30 min. Then, the cells were incubated overnight at 4 °C in BSA (3%) with the primary antibodies and were washed with PBS 3 times. Then, the cells were incubated for 60 min at 37 °C in BSA (3%) with the secondary antibodies against the pluripotency markers (Cellapy, Beijing, China). The nuclei were counterstained by DAPI, and the images were taken by an Olympus fluorescence microscope (BX51) (Olympus, Tokyo, Japan).

### Determination of karyotypes

The ADO2-iPSC lines were prepared for karyotyping by culturing the cells in medium containing 50 ng/mL colcemid for 6 h. The cells were digested with trypsin and were washed with PBS. Then, the cells were resuspended in 0.075 M KCl at 37 °C (30 min) for hypotonic treatment and were fixed in 3:1 methanol to acetic acid at room temperature (10 min). The fixing steps were repeated two times for 5 min. After the three washes with fixative, the cells were dropped on ice-cold slides, air dried at 75 °C (2 h), and stained by Giemsa using a standard G-banding technique.

### Detection of SeV genome and transgenes

The ADO2-iPSC lines were analyzed for SeV residues. The samples included the RNA that was left over from the reprogramming experiments; the ADO2-iPSC line and the H9 cell line were purchased from Cellapy Biotechnology (Beijing, China). The total RNA was extracted using TRIzol Reagent (Life Technologies). The cDNA was produced using a SuperRT cDNA Synthesis Kit (CW Biotech, Beijing, China). PCR was performed using a Taq MasterMix Kit (CW Biotech, Beijing, China) with the primers targets of SeV, KOS, KLF4, and c-MYC (Table 1) following the manufacturer’s instructions; electrophoresis of the PCR product was conducted with a 1% agarose gel at 120 V for 20 min. And the primer targets of SeV, KOS, KLF4, and c-MYC were designed according to the CytoTune™-iPS 2.0 Sendai Reprogramming kit USER GRIDE (Thermo Scientific).

### Pluripotency validation in vitro and in vivo

The ADO2-iPSC lines were cultured on plates that were coated with Matrigel in Urineasy Medium (Cellapy, Beijing, China) before the reprogrammed cells were tested for their capacity to spontaneously differentiate into the cells of all three germ layers. They were harvested when the confluency reached 50–80%, washed with PBS and treated with EDTA at 37 °C (5% CO_2_) for 3–5 min; the cells were collected and resuspended in PSCeasy Medium (Cellapy, Beijing, China) at 37 °C (5% CO_2_) for 30 min. Then, the supernatant was discarded, and the cells were resuspended in embryoid body (EB) differentiation medium, which was DMEM supplemented with 2 mM l-glutamine, 0.1 mM nonessential amino acids, 0.1 mM β-mercaptoethanol, and 20% FBS. The cells were seeded onto a 6-well plate for suspension culture for 7 days using EB differentiation medium. New medium was supplied every 48 h. Finally, the cells were harvested, and the RNA was isolated using TRNzol (TIANGEN, Beijing, China) and was transcribed into cDNA using the PrimeScript RT Reagent Kit (TaKaRa, Japan) following the manufacturer’s protocols. The cDNA primers of OCT4, GATA4, MSX1, SOX1, and GAPDH were used to analyze the specific gene expression of the germ layer by PCR (Table 1). The PCR program was set as follows: 94 °C for 2 min and 35 cycles of 94 °C for 30 s, 55 °C for 30 s, and 72 °C for 30 s. The final elongation was performed at 72 °C for 2 min. Electrophoresis of the PCR product was conducted with a 1.5% agarose gel at 100 V for 25 min.

To analyze the pluripotency in vivo, the ADO2-iPSCs that were maintained in the culture medium were harvested at 80% confluence and were resuspended in EDTA (0.5 mM), and centrifugated (1000 rpm) for 5 min. Then, the supernatant was discarded and the cells were resuspended in PBS. Then, the cells were injected into nonobese diabetic combined severe immunodeficient (NOD-SCID) mice by intramuscular injection. At 15 weeks post-injection, the mice were sacrificed and the tumors were excised. The tumor tissues were fixed in formalin (10%), embedded, sectioned, and finally stained by hematoxylin and eosin.

### Proteomic analysis

To characterize the ADO2-iPSCs by proteomics, peptides were prepared using the ADO2-iPSCs and normal control iPSCs (NC-iPSCs) that were induced from the urine of a healthy human donor and provided by Cellapy Biotechnology (Beijing, China). The NC-iPSCs were considered as a standard iPSC line with well-known characteristics, and our ADO2-iPSCs were generated using the same way. The protein profiling was performed as previously described methods [18]. In brief, the total protein levels were quantified by labeling peptides before being enriched with a TMT kit for 2-hydroxyisobutyryl. For K_hib_-modified peptide enrichment, fractionated peptides were dissolved in NETN buffer (100 mM NaCl, 1 mM EDTA, 50 mM Tris-HCl, 0.5% NP-40, pH 8.0) and incubated with prewashed antibody beads (Lot number: PTM804, PTM Bio, Hangzhou, China) at 4 °C overnight with gentle shaking. The beads were subsequently washed with NETN buffer four times and twice with H_2_O. The bound peptides were eluted from the beads with 0.1% trifluoroacetic acid. Finally, the eluted fractions were combined and vacuum-dried. For LC-MS/MS analysis, the resulting peptides were desalted with C18 ZipTips (Millipore) according to the manufacturer’s instructions. Lysine 2-hydroxyisobutyrylation quantification was conducted using spectral counting of the 2-hydroxyisobutyryl-enriched peptides. Detailed methods about the proteomic analysis were described in Additional file 10.

## Results

### Genotyping and the generation of ADO2-iPSCs

#### Genotyping of the osteopetrotic family

**Fig. 2:**
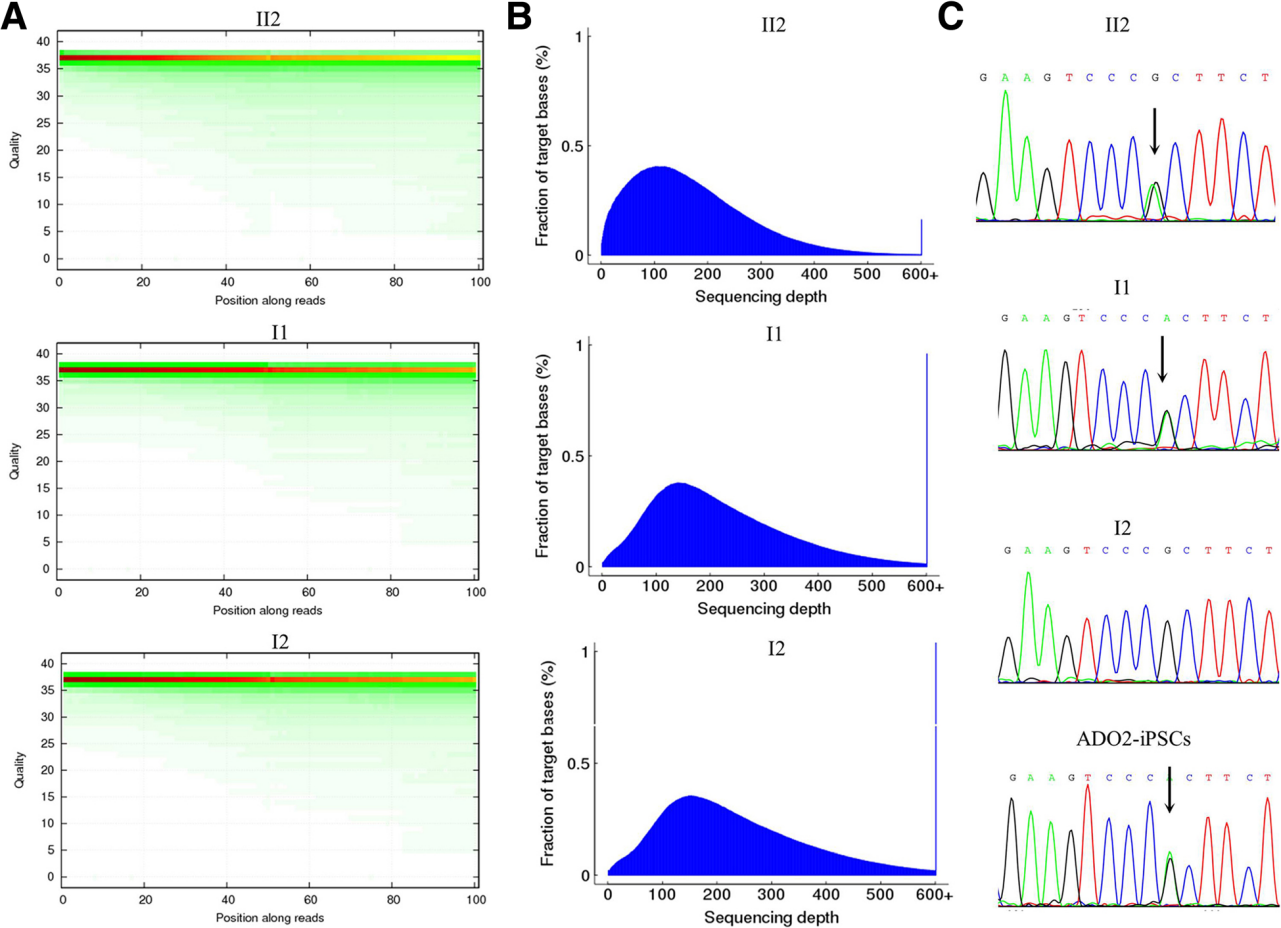
**a** The distribution of the base quality scores in the WES of the three samples, where the *X*-axis is the positions of the read, and the *Y*-axis is the quality value of the clean reads. **b** The distribution of per-base sequencing depth in the WES of the three samples. The *X*-axis denotes the sequencing depth, and the *Y*-axis indicates the percentage of total target regions under a given sequencing depth. **c** The mutation of CLCN7 (R286W) is confirmed by Sanger sequencing. And it is detected in the proband (II1), the father (I1), and the ADO2-iPSCs

The exomes of the proband and his parents from the ADO2 family were captured and sequenced. On average, 431.16 million clean reads were produced per sample, 99.72% of them were aligned to the human reference genome, and the average sequencing depth was 208.90× in the targeted exons (Tables 2 and 3). The quality of the sequencing data was good enough to perform further analysis (Fig. 2).

**Table 2:** Summary of the sequencing data

Samples	Raw reads	Raw bases (Mb)	Clean reads	Clean bases (Mb)	Clean data rate (%)	GC content (%)
II1	383,081,484	19,154.07	382,406,076	19,120.3	99.82	47.08
I1	425,798,332	21,289.92	424,425,506	21,221.28	99.68	46.80
I2	488,217,244	24,410.86	486,663,474	24,333.17	99.68	46.64

**Table 3:** Summary statistics of the alignments

Samples	Total effective reads	Total effective bases (Mb)	Effective sequences on target (Mb)	Mapping rate on genome (%)	Average sequencing depth on target
II1	308,239,198	15,339.07	8463.39	99.38	167.96
I1	336,634,204	16,784.23	11,134.21	99.65	220.96
I2	363,378,730	18,118.8	11,982.53	99.68	237.79

The detected DNA variants in the clean reads were compared to those in the NCBI dbSNP and 1000 Genomes Project databases. We found more than 95% of the genetic variations that we detected in the two databases (Table 4). All of the variants were then prioritized for further filtering by MAF, and we found 76,426, 78,144, and 77,889 rare variants with MAF < 1% in the proband, his father, and his mother, respectively. Considering that it was an inherited disease in one Chinese family, we focused on the shared rare variants in the affected individuals and reduced the variants to 3416 SNPs and 2649 InDels. Finally, we focused on the osteopetrotic genes that had been reported in the literature, and discovered a reasonable variant in CLCN7 (chr16:g.1506174G>A [NM_001287.5:c.856C>T, p. R286W]). It was a characterized mutation, and a study indicated that it could be found in more than 40% of osteopetrosis patients [19]; therefore, we believed this variant to be a candidate mutation.

**Table 4:** Summary statistics for the identified SNPs and InDels

Samples	Total SNPs	SNPs in dbSNP (%)	SNPs in 1KG (%)	SNPs in ESP6500 (%)	MAF of SNPs ≤ 0.01	Total InDels	InDels in dbSNP (%)	InDels in 1KG (%)	InDels in ESP6500 (%)	MAF of InDels ≤ 0.01
II1	99,250	98.29	95.66	37.03	6483	14,130	82.31	61.97	14.37	5207
I1	97,674	98.10	95.35	36.54	6709	16,160	78.24	56.58	12.36	6850
I2	98,849	98.13	95.49	37.29	6641	16,292	78.28	57.06	13.10	6849

Note: *ESP* NHLBI Grand Opportunity Exome Sequencing Project, *MAF* minor allele frequency

To confirm the findings of WES, we tested the candidate mutation (R286W) in CLCN7 in the family members by a combination of PCR and Sanger sequencing. As shown in Fig. 2, we found two radiographically affected members including the proband and his father, who were heterozygous for the mutation. The other healthy family members and the 30 population-matched controls did not carry the mutation.

#### Generation of ADO2-iPSCs from the proband

**Fig. 3:**
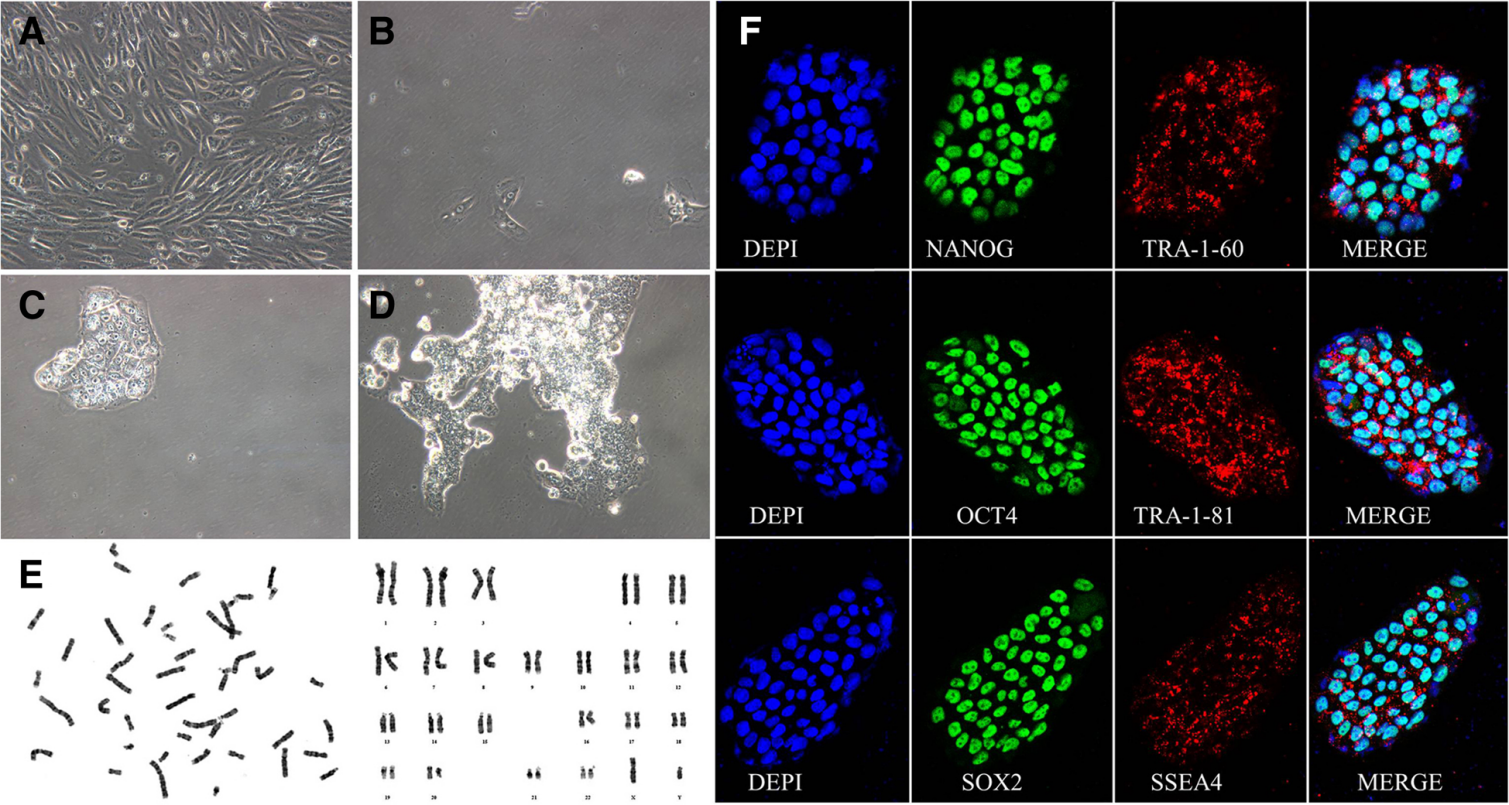
Generation of ADO2-iPSCs from urinary cells. **a** Urinary cells are cultured and amplified before plasmid infection. **b** Small cell colonies appear on day 5 after plasmid infection. **c** The cell colony appears on day 8 after plasmid infection. **d** The growing cell colony appears on day 17 after plasmid infection. **e** G-banding detection indicates the ADO2-iPSCs with a normal male karyotype. **f** Immunofluorescence staining indicates the ADO2-iPSCs expressing typical pluripotent markers, such as NANOG, TRA-1-60, OCT4, TRA-1-81, SOX2, and SSEA-4

We collected urine cells from the proband and cultured them with steady proliferation for one passage. We transfected urine cells with SeV encoding OCT3/4, SOX2, KLF4, and c-MYC and found that human embryonic stem cell-like colonies first appeared 5 to 8 days after infection. We then chose the large, typical human embryonic stem cell-like colonies to expand at passage 3 (Fig. 3). The STR profiling confirmed that the ADO2-iPSCs carried identical STR profiles as those from the ADO2-blood taken from the proband (Table 5).

**Table 5:** Short tandem repeat (STR) profiling from ADO2-iPSCs and ADO2-Blood taken from the proband confirmed the same genetic identity

Allele	ADO2-iPSCs	ADO2-Blood
D3S1358	15, 18	15, 18
D1S1656	14, 18.3	14, 18.3
D6S1043	14, 18	14, 18
D13S317	11, 12	11, 12
Penta E	11, 21	11, 21
D16S539	9, 10	9, 10
D18S51	16, 17	16, 17
D2S1338	17, 19	17, 19
CSF1PO	10, 11	10, 11
Penta D	9, 10	9, 10
TH01	9, 9.3	9, 9.3
vWA	14, 17	14, 17
D21S11	29, 30	29, 30
D7S820	11, 11	11, 11
D5S818	11, 12	11, 12
TPOX	9, 11	9, 11
D8S1179	13, 15	13, 15
D12S391	18, 19	18, 19
D19S433	13, 15	13, 15
FGA	22, 24	22, 24
AMEL	X, Y	X, Y

#### General characteristics of the ADO2-iPSCs

**Fig. 4:**
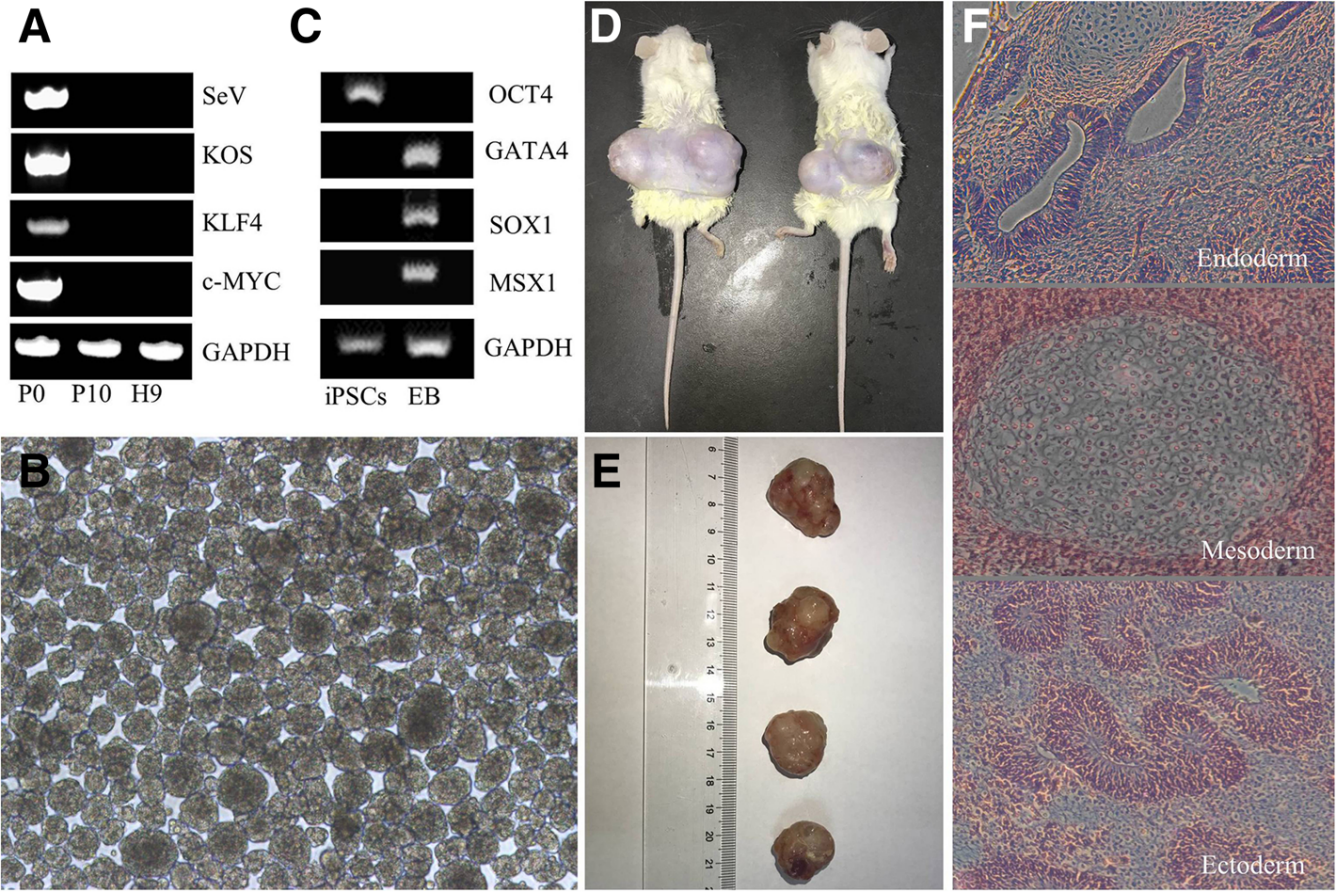
Detection of gene expression by PCR. **a** The picked ADO2-iPSCs on day 17 after plasmid infection (passage number = 0, P0) are positively expressed with SeV genome and transgenes; the passage ADO2-iPSCs ((passage number = 10, P10) are similar to the H9 cell lines, and they are negative for the SeV genome and transgenes. **b**, **c** EB formation was generated from the ADO2-iPSCs, and the marker genes, including GATA4, SOX1, and MSX1, were positively expressed. The grouping of gels was cropped from different parts of the same gel and from the full-length agarose gel, which are presented in Additional file 1: Figure S1. **d** Teratoma formation in the backs of NOD-SCID mice after 8 weeks of the injection with ADO2-iPSCs. **e** The teratomas taken from the mice. **f** Typical histology of the teratomas with all three germ layers (endoderm, mesoderm, and ectoderm)

To analyze the stemness of the urine-derived ADO2-iPSCs, we performed immunostaining and found positive expressions of NANOG, TRA-1-60, OCT4, TRA-1-81, SOX2, and SSEA4 (Fig. 3). We also found that alkaline phosphatase is positively expressed in ADO2-iPSCs. We found a normal karyotype of 46, XY (Fig. 3) in the ADO2-iPSCs and confirmed that the cell line carried the same mutation, CLCN7 (R286W), which was previously discovered in the patient genome (Fig. 2). To test for the residual SeV in the ADO2-iPSCs, we performed PCR and electrophoresis analyses and found that the early passage ADO2-iPSCs (passage number = 0, P0) positively expressed SeV, KOS, Klf4, and c-Myc and that the ADO2-iPSCs with a high passage number (passage number = 10, P10) negatively expressed the transduced transgenes (Fig. 4).

#### Potential function of the ADO2-iPSCs

To examine the differentiation potential of ADO2-iPSCs in vitro, we tested for EB formation spontaneously from ADO2-iPSCs in a suspension culture. EBs were clearly visible after 7 days in suspension (Fig. 4). We isolated the total RNA of cells and found that the lineage-specific genes of OCT4 were only negatively expressed, while GATA4, MSX1, and SOX1 were positively expressed in the differentiated cells. For the test of pluripotency in vivo, we transplanted the ADO2-iPSCs into two NOD-SCID mice and found the formation of teratomas 8 weeks following the injection. We found that the teratomas had derivatives of all three germ layers, such as the neural tube differentiated from the ectoderm, the endogland differentiated from the endoderm, and the cartilage differentiated from the mesoderm (Fig. 4).

### Whole-cell proteomic profiling of the ADO2-iPSCs

**Fig. 5:**
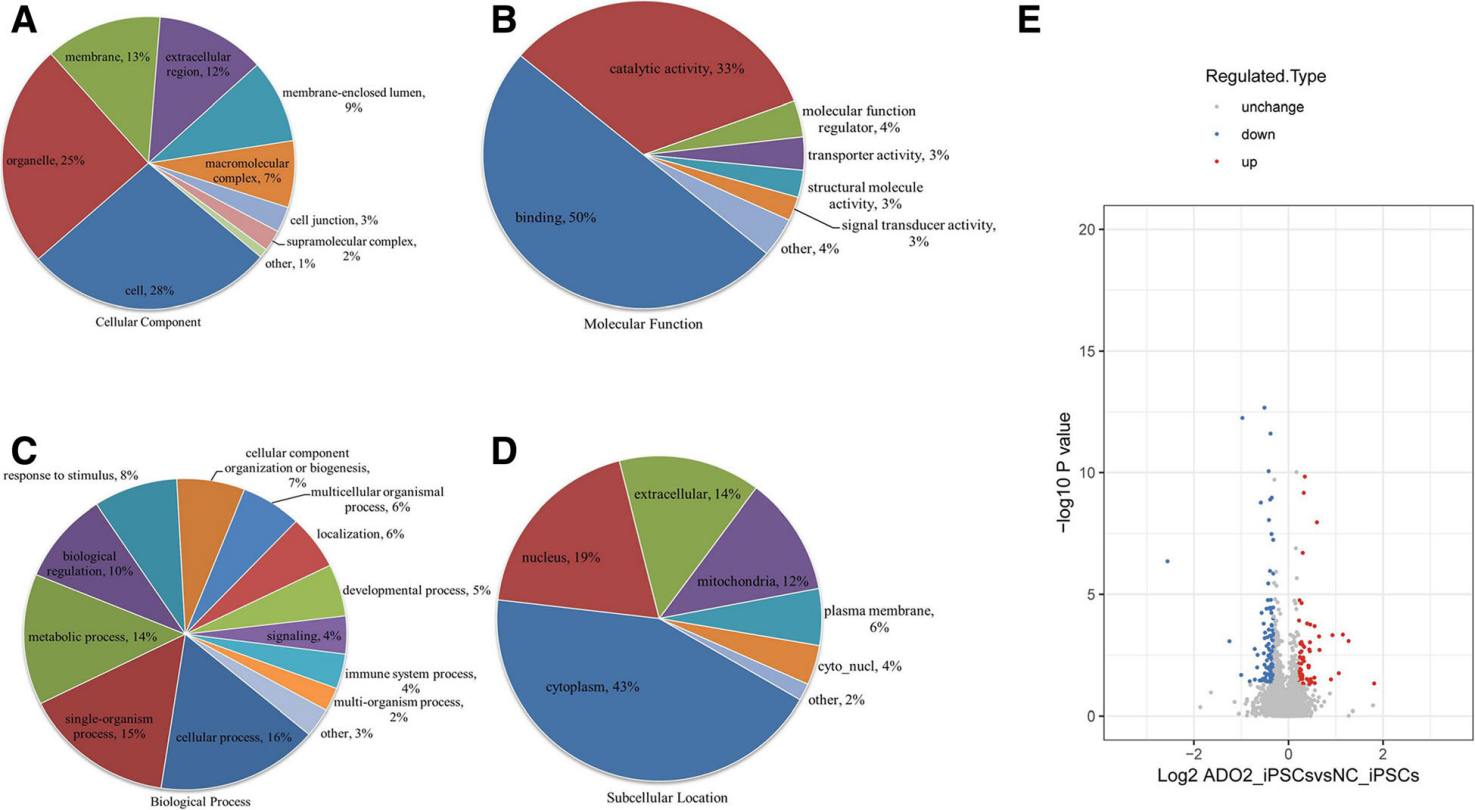
Classifications of the identified proteins in the ADO2-iPSCs. **a** The cellular component classifications. **b** The molecular function classifications. **c** The biological processes classifications. **d** Subcellular localization of the identified proteins. **e** Volcano plot of the differentially expressed proteins in the ADO2-iPSCs

Totally, 7405 proteins were identified, among which 6536 proteins were with a quantifiable level between the ADO2-iPSCs and NC-iPSCs. To check our MS data, the quality control was performed, and our results indicated that our MS data satisfied the subsequent advanced analysis (Additional file 2: Figure S2). Further bioinformatic analysis for 6536 quantifiable proteins have shown that these proteins were localized in the cytoplasm and nucleus and extracellularly and were then further classified by gene ontology (GO) annotation (Fig. 5). In the quantifiable proteins, we found 6359 proteins (97.3%) were expressed at a similar level between the two different cell lines. The similarities included a number of pluripotency markers (Additional file 9: Table S1) [20, 21]. And according to a fold change of more than 1.2 or less than 1/1.2 and *P* < 0.05, we identified only 177 differentially expressed proteins (DEPs) (Table 6). Among these DEPs, 70 were upregulated and 107 were downregulated (Fig. 5). Then, we further gathered the DEPs to conduct GO, KEGG pathway, and protein domain enrichment and clustering analysis and found that their functions were multifarious (Additional file 3: Figure S3, Additional file 4: Figure S4, Additional file 5: Figure S5, and Additional file 9: Tables S2, S3, S4). Interestingly, the upregulated protein ISG15 (2.305 fold change, *P* = 0.00046) was involved in bone formation [22] and highly enriched in the RIG-I-like receptor signaling pathway, which may have a close relationship with the disease of osteopetrosis (Additional file 6: Figure S6).

**Table 6:** Differentially expressed protein summary (only top 10 proteins were listed)

Index	Protein accession	Protein description	Gene name	Up/down	Fold change	*P* value
1	Q96LJ7	Dehydrogenase/reductase SDR family member 1 OS=Homo sapiens OX=9606 GN=DHRS1	DHRS1	Up	3.646	0.046916
2	Q9UII4	E3 ISG15--protein ligase HERC5 OS=Homo sapiens OX=9606 GN=HERC5	HERC5	Up	2.506	0.00084139
3	P05161	Ubiquitin-like protein ISG15 OS=Homo sapiens OX=9606 GN=ISG15	ISG15	Up	2.305	0.00045779
4	Q10589	Bone marrow stromal antigen 2 OS=Homo sapiens OX=9606 GN=BST2	BST2	Up	2.169	0.0177766
5	Q5T6V5	Queuosine salvage protein OS=Homo sapiens OX=9606 GN=C9orf64	C9orf64	Up	1.978	0.00048022
6	Q16850	Lanosterol 14-alpha demethylase OS=Homo sapiens OX=9606 GN=CYP51A1	CYP51A1	Down	0.833	0.00041878
7	P21399	Cytoplasmic aconitate hydratase OS=Homo sapiens OX=9606 GN=ACO1	ACO1	Down	0.831	0.030976
8	P48147	Prolyl endopeptidase OS=Homo sapiens OX=9606 GN=PREP	PREP	Down	0.831	1.3993E−06
9	P08069	Insulin-like growth factor 1 receptor OS=Homo sapiens OX=9606 GN=IGF1R	IGF1R	Down	0.83	0.023502
10	P28838	Cytosol aminopeptidase OS=Homo sapiens OX=9606 GN=LAP3	LAP3	Down	0.83	0.000098074

### Proteome-wide lysine 2-hydroxyisobutyrylation of the ADO2-iPSCs

#### Characterization of K_hib_-modified proteins in the ADO2-iPSCs

**Fig. 6:**
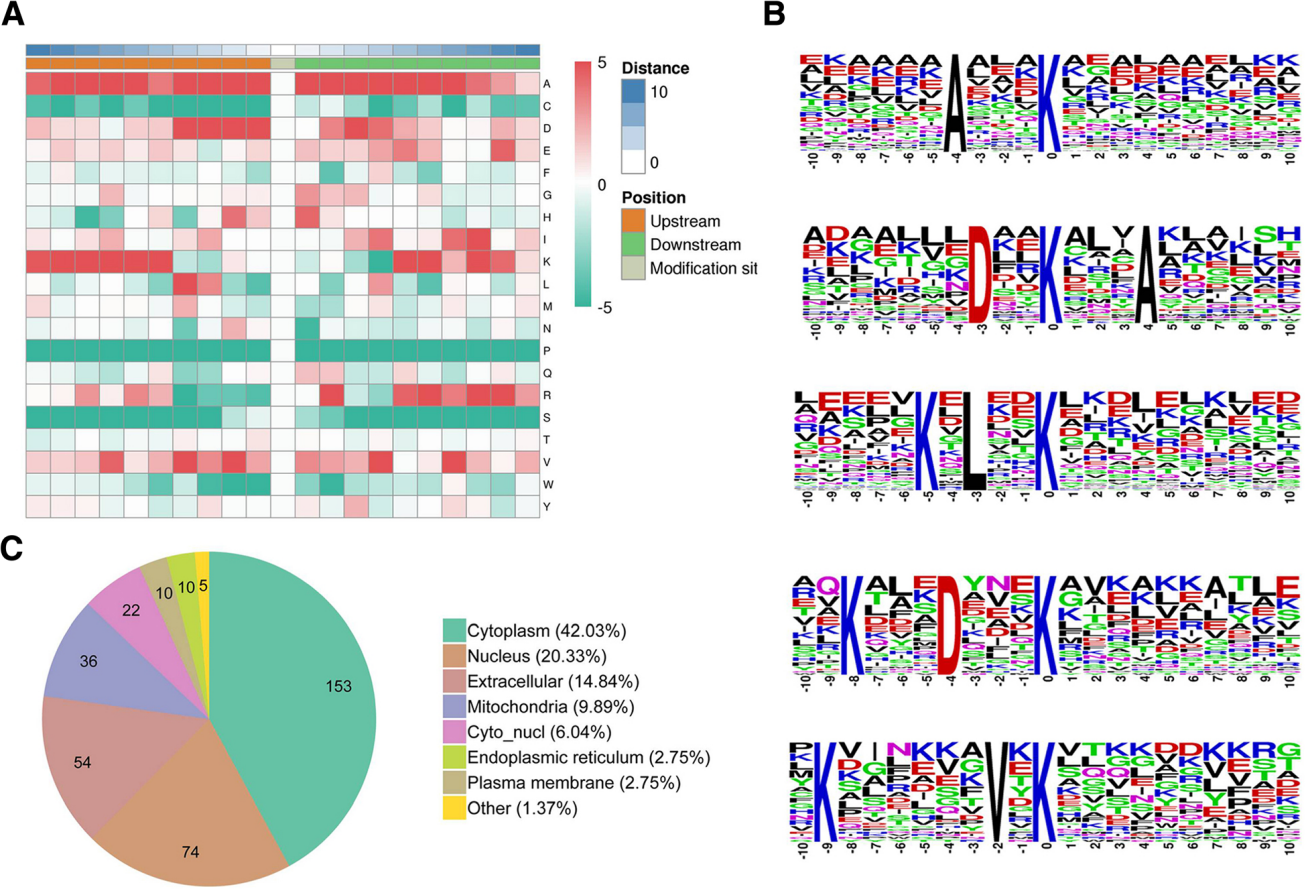
Properties of all the identified K_hib_-modified peptides in the ADO2-iPSCs. **a** Heat map of the amino acid compositions of the lysine 2-hydroxyisobutyrylation sites showing the frequency of different types of amino acids around 2-hydroxyisobutyrylated lysine. **b** The subcellular localization of the K_hib_-modified proteins. **c** The top 5 strikingly 2-hydroxyisobutyrylation motifs and conservation of 2-hydroxyisobutyrylation sites are shown (motif score > 15.00)

**Fig. 7:**
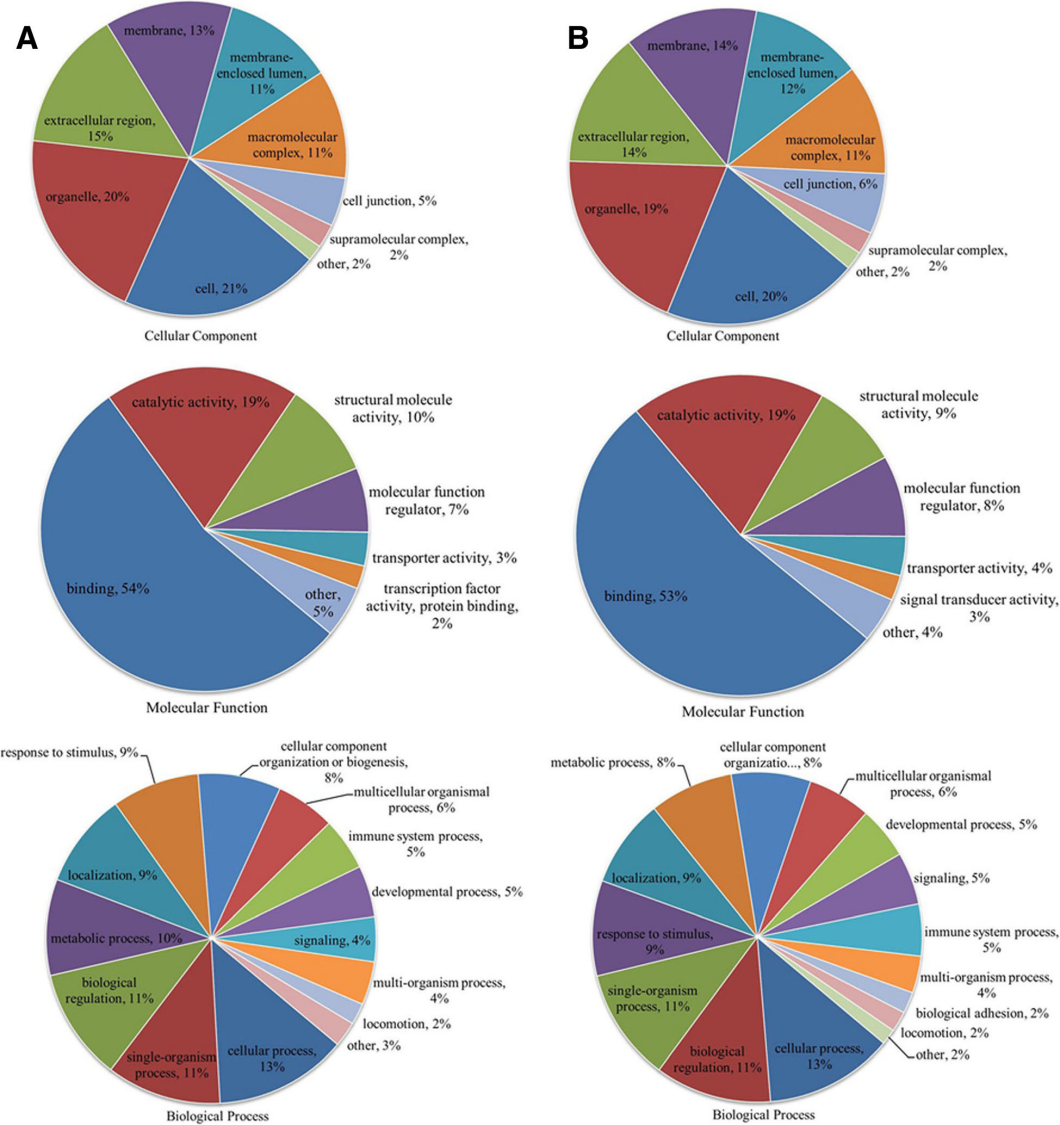
GO classifications of the K_hib_-modified proteins in the ADO2-iPSCs. **a** the GO classifications of upregulated proteins. **b** the GO classifications of downregulated proteins

Of all the 4327 peptides acquired, 3664 peptides in 1036 proteins were identified with K_hib_ modifications, among which 897 K_hib_-modified proteins were with a quantifiable level between the ADO2-iPSCs and NC-iPSCs. Intensive sequence motif analysis for the 3664 K_hib_-modified peptides was carried out, and 14 conserved motifs were identified. Especially, the motifs Axxx_K_, Dxx_K_xxxA, KxLxx_K_, KxxxDxxx_K_ and KxxxxxxVx_K_ (Motif Score > 15.00) were strikingly conserved. Hierarchical cluster analysis for these motifs demonstrated that the enrichment of charged A residues was observed in the + 5 to − 5 positions, representing a feature of K_hib_ in ADO2-iPSCs (Fig. 6). Further advanced analysis for 897 quantifiable K_hib_-modified proteins has shown that these proteins were distributed in the cytoplasm and nucleus and extracellularly, and associated with different kinds of biology functions (Figs. 6 and 7). According to a fold change of more than 1.2 or less than 1/1.2 and *P* < 0.05, we identified 410 differentially expressed K_hib_-modified proteins (Table 7), of which, 216 were upregulated and 194 were downregulated.

**Table 7:** Differentially K_hib_-modified protein summary (only top 10 proteins were listed)

Index	Protein accession	Protein description	Gene name	Up/down	Fold change	*P* value
1	P07996	Thrombospondin-1	THBS1	Up	2.871	0.00279623
2	Q86UX7	Fermitin family homolog 3	FERMT3	Up	2.604	2.79667E−06
3	P25311	Zinc-alpha-2-glycoprotein	AZGP1	Up	2.322	0.000392084
4	P62826	GTP-binding nuclear protein Ran	RAN	Up	2.118	0.00266401
5	O75368	SH3 domain-binding glutamic acid-rich-like protein	SH3BGRL	Up	2.03	0.00362243
6	P16949	Stathmin	STMN1	Down	0.833	0.00219361
7	P55010	Eukaryotic translation initiation factor 5	EIF5	Down	0.833	0.0160196
8	P78347	General transcription factor II-I	GTF2I	Down	0.833	0.00132784
9	Q9Y678	Coatomer subunit gamma-1	COPG1	Down	0.833	0.041308
10	P78371	T-complex protein 1 subunit beta	CCT2	Down	0.832	8.6561E−06

#### Functional enrichment and clustering analysis of the differentially K_hib_-modified proteins in the ADO2-iPSCs

**Fig. 8:**
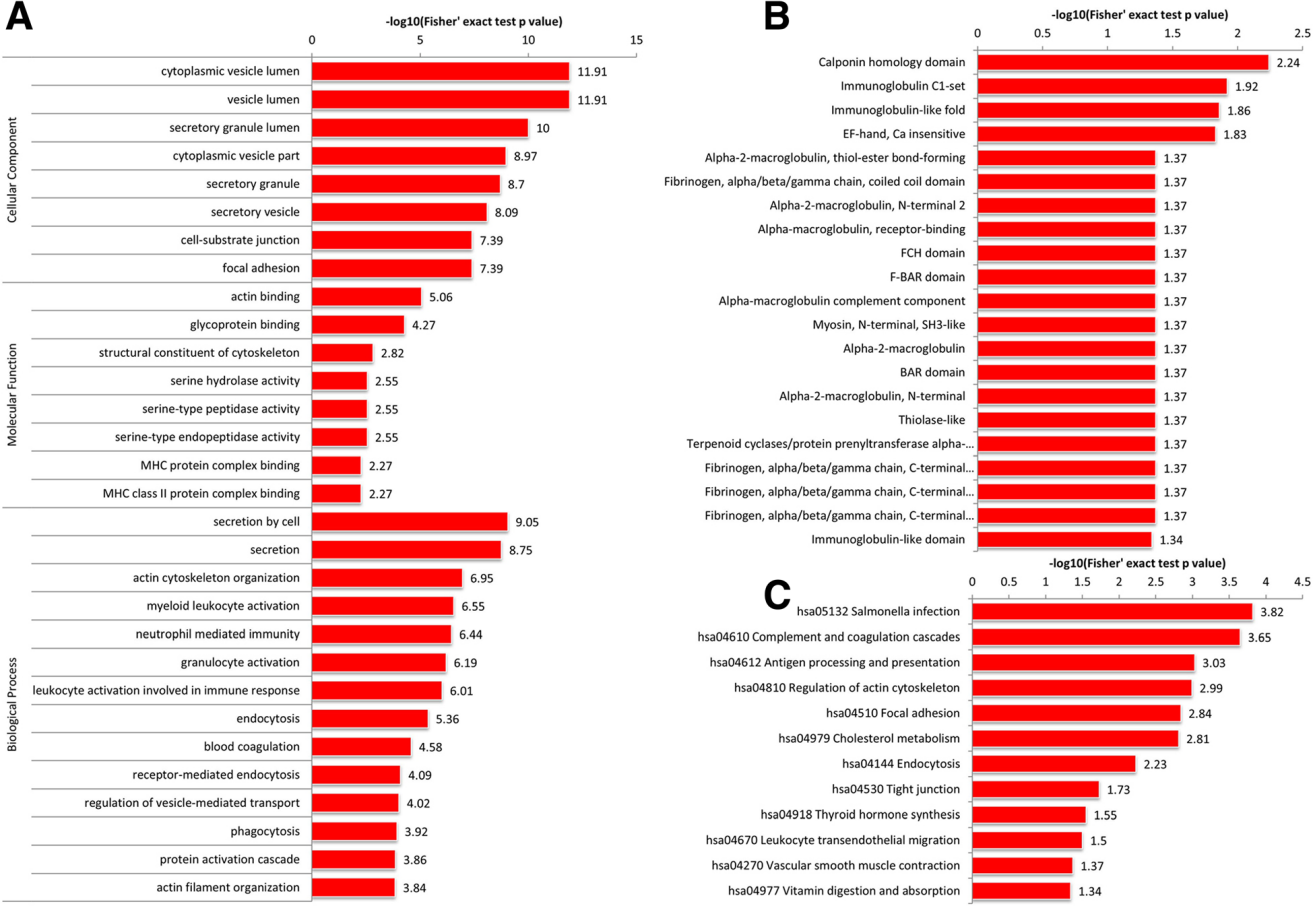
Functional enrichment analysis of the K_hib_-modified proteins in the ADO2-iPSCs. **a** GO-based functional enrichment analysis. **b** Protein domain functional enrichment analysis. **c** KEGG-based functional enrichment analysis

We gathered the 410 differentially proteins with 629 K_hib_-modified sites to conduct GO, KEGG pathway, and protein domain functional enrichment analysis and found that their functions were diversiform (Fig. 8 and Additional file 9: Tables S5, S6, S7), such as the 30 GO terms, and 12 significantly pathways and 21 protein domains were significantly enriched in the ADO2-iPSCs. Then, we divided the differentially K_hib_-modified proteins into four quantiles (Q1–Q4) according to fold changes: Q1 (0 < ratio < 0.77), Q2 (0.77 < ratio < 0.83), Q3 (1.2 < ratio < 1.3), and Q4 (ratio > 1.3), and further performed functional enrichment clustering analysis (Additional file 7: Figure S7 and Additional file 8: Figure S8). GO enrichment-based clustering analysis showed that the differentially K_hib_-modified proteins in Q1 were mainly enriched in actin binding, receptor binding, and iron ion binding, while the GO terms related to actin binding, glycoprotein binding, and structural molecule activity were mainly enriched in Q4. For KEGG functional enrichment clustering analysis, we found that the complement and coagulation cascades, malaria, and porphyrin and chlorophyll metabolism were the most prominent pathways enriched in Q1, while the salmonella infection was the vitally important pathway in Q4. In addition, for the protein domain functional enrichment clustering analysis, the differentially K_hib_-modified proteins in Q1 were clustered in fibrinogen, alpha/beta/gamma chain, and coiled coil domain, and the differentially expressed K_hib_-modified proteins in Q4 were most significantly enriched in sushi/scr/ccp domain, immunoglobulin-like fold, and immunoglobulin-like domain.

### The potential relationships between DEPs, K_hib_-modified proteins, and ADO2

**Fig. 9:**
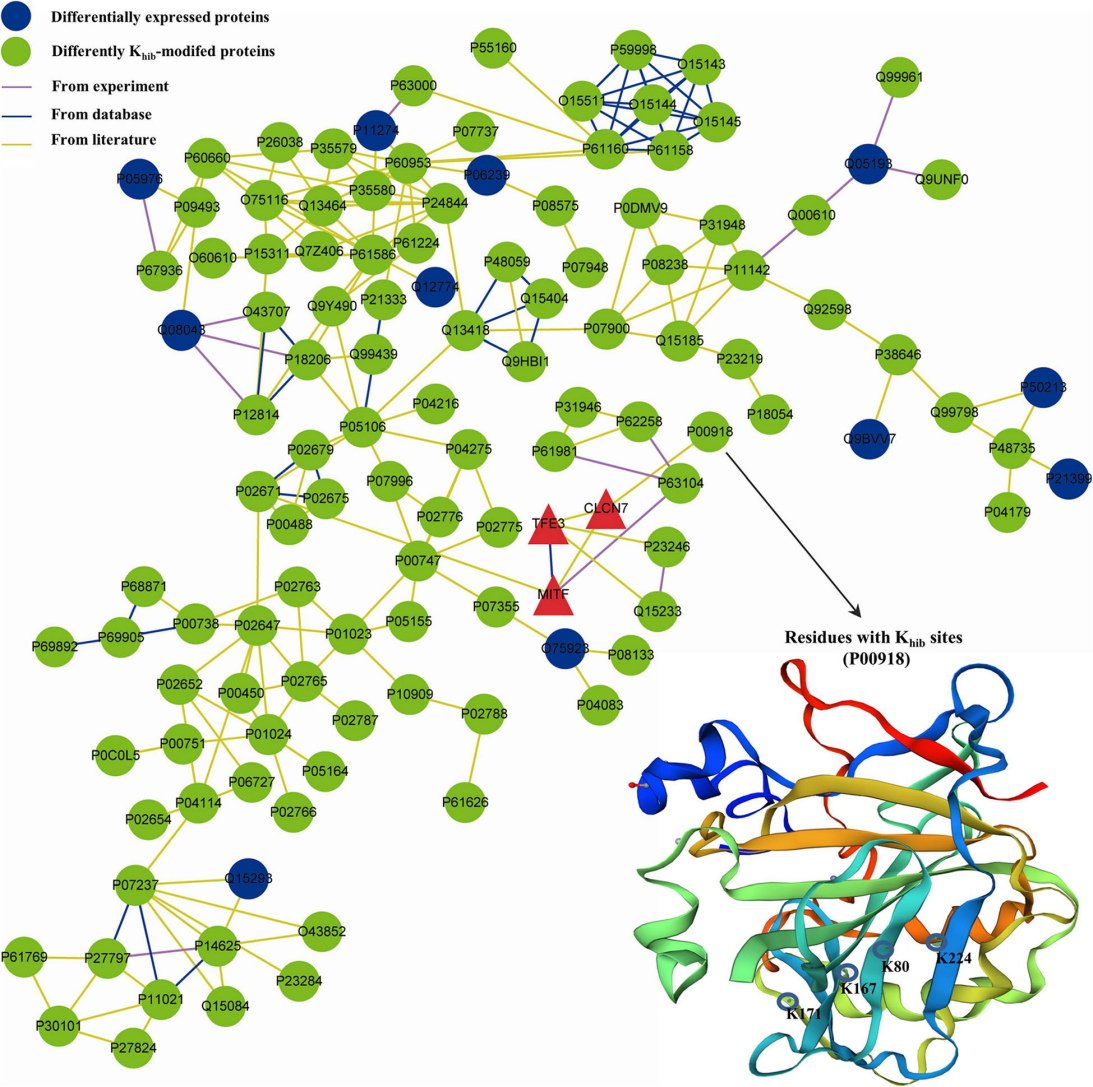
The potential relationships between the DEPs, the differently K_hib_-modified proteins, MITF, TFE3, and CLCN7 in the ADO2-iPSCs (the red triangles represent ClCN7, MITF, and TFE3. The purple circles represent the DEPs, and the green circles represent the differently K_hib_-modified proteins); and the three-dimensional structure of K_hib_-modified protein (P00918, Carbonic anhydrase 2) is shown, which includes the four K_hib_ sites [25]

The ADO2-iPSCs were carrying the disease-causing mutation in CLCN7, which had been identified as a putative target of MITF and TFE3 [23]. Therefore, the direct or indirect relationship among the DEPs, the K_hib_-modified proteins, and three genes may be associated with ADO2. In order to explore their potential relationship, we try to construct a network of protein-protein interactions (PPIs) by STRING [24]. The interaction network form STRING was visualized by Cytoscape 3.6.1., and our data indicate that some close relationships among the DEPs, K_hib_-modified proteins, and ADO2 could be found from experiments, databases, or literature; for example, we could find direct relationships between CLCN7/MITF/TFE3 and K_hib_-modified proteins, such as P00747 (PLG), P63104 (YWHAZ), Q15233 (NONO), P23246 (SFPQ), and P00918 (carbonic anhydrase 2, CA2) (Fig. 9) [25].

## Discussion

Osteopetrosis is an inherited disease, and the identification of the genetic variants and the generation of iPSCs with the underlying phenotype may be valuable for personalized medicine. However, more than 20 genes have been reported to be associated with osteopetrosis, and it is still a challenge to analyze all of the osteopetrotic genes by traditional tools. Therefore, we performed WES for genotyping because this kind of technology has the ability to capture and analyze almost all protein-coding genes. It is a high-throughput approach, and it may be a challenge to understand the great number of DNA variants when the sequencing depth is increasing. In this study, we used the 1000 Genomes Project and NHLBI Grand Opportunity Exome Sequencing Project databases to filter the variants, and thousands of shared variants remain in the proband and his father. This strategy may be useful to decrease the quantity of variants, but it remains a challenge to reveal the disease-associated mutation. The family may have the disease due to a previously associated mutation rather than a novel gene [1]. Therefore, we focused on the known genes that result in osteopetrosis and found CLCN7 (chr16:g.1506174G>A) as a candidate mutation. The candidate DNA mutation may cause defects in translations of ClC-7; the affected amino acid (R286) is conserved among ClC chloride channel family, and it is located outside the transmembrane domain [26]. Some studies have documented that the chloride channel acts as the Cl−/H+ exchanger, which is regulated by a voltage-gating mechanism, and plays a very important role in the acidification of osteoclast-mediated degradation of bone tissue; mutations in CLCN7 may be responsible for various types of osteopetrosis [27, 28]. The severity of CLCN7-associated osteopetrosis is diverse, and the symptoms may range from asymptomatic to mild in ADO2 patients and may even be ARO with a very severe phenotype [29]. CLCN7 (R286W) is a known mutation of ADO2 that can be found in ADO2 patients from China and other nations [30, 31]. In this study, the mutation found by WES is confirmed by Sanger sequencing, and it is absent in the healthy family member and in the controls. Therefore, we considered CLCN7 (R286W) with genotype-phenotype correlations to be the disease-causing mutation of the ADO2 family.

In the clinic, bone marrow transplantation has been performed as therapy to treat many kinds of ARO, but there is currently no effective treatment for ADO2 [32]. Therapy for patients with ADO2 is commonly palliative, such as fracture repairs, decompression of the nerves, and pain control; this is partly due to the lack of proper ADO2 animal models and cost-effective bone marrow from donors [33]. Therefore, the generation of animal disease models and cell models in vitro combined with the ability to modify mutations may be valuable not only for drug discovery but also to elucidate the mechanisms and treatment for this kind of disease [34]. Fortunately, the first mouse model of ADO2, which carried a heterozygous mutation (p.G213R) in the Clcn7 gene, was generated in 2014 [33]. Recently, some studies indicate that iPSCs provide a relatively noninvasive way to study the cell types affected by human diseases from clinical patients; therefore, they may act as a bridge between the clinic and bench research [35–37]. Since iPSC technology has been established, iPSC lines have been developed for patients with neurodegenerative, metabolic, and immune disorders [38–40]. Recently, clinically relevant disease-specific iPSCs were also successfully generated from osteopetrotic mouse with Tcirg1 mutation and ARO patient with CLCN7 mutation, and they seem to be ideal cell source for translational researches, because these cell lines were carrying identical genetic background as the donors and pluripotency [38, 41]. Therefore, iPSCs generated from ADO2 patients may be a perfect way to model this kind of inherited disease. However, no ADO2-specific iPSCs have been developed and well characterized.

For pharmaceutical and clinical applications, somatic cells, such as fibroblasts, bone marrow cells, and epithelial cells, may be used as sources to generate iPSCs by introducing SOX2, OCT3/4, c-MYC, and KLF4 or SOX2, NANOG, OCT3/4, and LIN28 [42]; in practice, we should consider the way that somatic cells obtain mutations and their differentiation propensities [34]. Our previous study has indicated that urine cells can be obtained by noninvasive procedures and observed with high efficiency of reprogramming [40]. Therefore, we preferred to generate ADO2-iPSCs from urine, which carry identical STR profiles and the ClCN7 (R286W) mutation as those from the blood taken from the proband in this study. Our results indicate that the somatic cells obtained from the patient are simple and accessible. Some studies have indicated that human iPSCs could be generated by the reprogramming method using either lentiviruses or retroviruses to deliver transgenes [43]; this kind of reprogramming method may bring insertions of viral transgenes to the host genome, and the safety of the generated iPSCs may still be a problem for clinical applications [34]. Therefore, we chose SeV vectors (cytoplasmic RNA vector) to deliver transgenes into urine cells to generate ADO2-iPSCs. Our results indicated that SeV is one class of gene transfer vectors that has a high transduction efficiency without viral genomic integration. Furthermore, ADO2-iPSCs exhibit typical embryonic stem cell morphology, such as the positive expression of pluripotency markers, including NANOG, TRA-1-60, OCT4, TRA-1-81, SOX2, and SSEA4; their karyotypes are normal; and they have the ability to form EBs in vitro and teratomas in vivo. These biology characteristics of ADO2-iPSCs generated from urine cells are similar to the ARO patient-specific iPSCs derived from mesenchymal stromal cells [41]. Furthermore, proteomic analysis has been found to be a valuable way to define and characterize iPSCs [44]. In our proteomic profiling, we detected thousands of proteins, and majority of them (97.3%) were expressed at a similar level between the two different cell lines. These proteins included some common pluripotency markers, such as POU5F1, SOX2, SSEA4, and LIN28. All of these data indicate that our ADO2-iPSCs are successfully generated.

Some studies have indicated that proteomic changes affecting cellular processes in human disease would be present in the undifferentiated iPSCs generated from the patient’s somatic cells [10, 11]. Therefore, we attempt to perform high-resolution LC-MS/MS and bioinformatics analysis for the identification of the differently expressed and modificated proteins that have been previously known associating with ADO2. In the present study, the whole peptides and the K_hib_-modified peptides captured by antibody-based affinity enrichment of the ADO2-iPSCs and NC-iPSCs, were analyzed by our proteomic approaches respectively. Comparing with DEPs, we discover that there is a higher proportion of differently K_hib_-modified sites in the ADO2-iPSCs. These data indicate that the DEPs and K_hib_-modified proteins involve widely biology functions, and further identification of protein-protein interactions (PPIs) may be valuable for us to reveal some proteins previously known associating with ADO2 [45, 46].

In our study, we constructed a network of PPIs using STRING, which is an important database for prediction protein function and constructed network of PPIs [47, 48]. By this way, we can find the potential relationship between different proteins (genes) visually. Interestingly, we can find that there is one direct relationship between CLCN7 and the K_hib_-modified proteins (P00918, CA2) from the network. This protein is also one of the DEPs and K_hib_-modified proteins, which were significantly enriched in the categories of the protein binding and catalytic activity in our biology function analysis. Some studies have indicated that carbonic anhydrase 2 (CA2) defect would cause a series symptoms, including osteopetrosis with renal tubular acidosis and brain calcification [49]. And molecular evidences confirm that CA2 played important roles in ion transport and pH regulation in several organisms and CA2 deficiency would interfere with osteoclast functions [50]. In the present study, we can find four differently K_hib_-modified sites in CA2. And two of them, such as K80 and K224, are located at beta strand and alpha helix respectively. These modified sites may affect the structure and enzymatic activity of CA2. Although further experimental evidences are needed, these results indicated that K_hib_-modified proteins may be some novel interesting events associated with osteopetrosis.

## Conclusion

In summary, we have successfully genotyped an autosomal dominant osteopetrosis family and generated ADO2-iPSCs with the known mutation CLCN7 (R286W) from the urine cells of ADO2 patients. Our results provide new insights into ADO2-iPSCs with known mutation CLCN7(R286W) based on whole-cell proteome and lysine 2-hydroxyisobutyrylated analyses. The transgene, integration free ADO2-iPSCs with the characteristics of multiple potentiality and lysine 2-hydroxyisobutyrylation may serve as a cell model for the preclinical trials of ADO2. Our future work may focus on the mutation collection and reveal its side effects, which may be valuable for future therapeutic use of the ADO2-iPSCs.

## Additional files

Additional file 1: **Figure S1.** Uncropped and full-length gels. The displayed gels correspond to the following figures of the main text: (a) Fig. 4a. (b) Fig. 4b. (JPG 337 kb)

Additional file 2: **Figure S2.** Quality control of the MS data. (A). The length distribution of the identified peptide. (B). Relationship between the identified protein mass and coverage. (C). Mass precision distribution of MS data. (JPG 438 kb)

Additional file 3: **Figure S3.** Functional enrichment analysis of the DEPs in the ADO2-iPSCs. (A). GO-based functional enrichment analysis. (B). KEGG-based functional enrichment analysis. (C). Protein domain enrichment analysis. (JPG 833 kb)

Additional file 4: **Figure S4.** GO functional enrichment clustering analysis of the DEPs of ADO2-iPSCs. All of the DEPs were divided into four quantiles (Q1–Q4) according to fold changes: Q1 (0 < ratio < 0.77), Q2 (0.77 < ratio < 0.83), Q3 (1.2 < ratio < 1.3), and Q4 (ratio > 1.3), and further performed GO, KEGG pathway and protein domain functional enrichment clustering analysis. (A). Biological process. (B). Cellular component. (C). Molecular function. (JPG 1015 kb)

Additional file 5: **Figure S5.** KEGG pathway (A) and protein domain (B) functional enrichment clustering analysis of the DEPs in ADO2-iPSCs. (JPG 1117 kb)

Additional file 6: **Figure S6.** The KEGG RIG-I-like receptor signaling pathway (red represents upregulated, green represents downregulated, yellow indicates that there are multiple proteins in this node, including differentially upregulated and downregulated proteins.). (JPG 200 kb)

Additional file 7: **Figure S7.** GO functional enrichment clustering analysis of the differently K_hib_-modified proteins in ADO2-iPSCs. All of the differently K_hib_-modified proteins were divided into four quantiles (Q1–Q4) according to fold changes: Q1 (0 < ratio < 0.77), Q2 (0.77 < ratio < 0.83), Q3 (1.2 < ratio < 1.3), and Q4 (ratio > 1.3), and further performed GO, KEGG pathway and protein domain functional enrichment clustering analysis. (A). Biological process. (B). Cellular component. (C). Molecular function. (JPG 930 kb)

Additional file 8: **Figure S8.** KEGG pathway (A) and Protein domain (B) functional enrichment clustering analysis of the differently K_hib_-modified proteins in the ADO2-iPSCs. (JPG 963 kb)

Additional file 9: **Table S1.** Proteomic analysis revealed the similar expression of pluripotency markers in the ADO2-iPSCs and NC-iPSCs. Table S2 GO functional enrichment analysis of the DEPs of the ADO2-iPSCs. Table S3 KEGG functional enrichment analysis of the DEPs of the ADO2-iPSCs. Table S4 Protein domain functional enrichment analysis of the DEPs of the ADO2-iPSCs. Table S5 GO functional enrichment analysis of the differently K_hib_-modified proteins of the ADO2-iPSCs. Table S6 KEGG functional enrichment analysis of the differently K_hib_-modified proteins of the ADO2-iPSCs. Table S7 Protein domain functional enrichment analysis of the differently K_hib_-modified proteins of the ADO2-iPSCs. (XLSX 32 kb)

Additional file 10: Full proteomic analysis methods. (DOCX 21 kb)

## Data Availability

Please contact author for data requests.

## Abbreviations

ADO2-iPSCs: Autosomal dominant osteopetrosis type II-specific induced pluripotent stem cells
ARO: Autosomal recessive osteopetrosis
CA2: Carbonic anhydrase 2
DEPs: Differentially expressed proteins
GO: Gene ontology
HSCT: Hematopoietic stem cell transplantation
KEGG: Kyoto Encyclopedia of Genes and Genomes
K_hib_: 2-Hydroxyisobutyrylation
NC-iPSCs: Normal control iPSCs
STRING: Search tool for the retrieval of interacting genes/proteins
TMT: Tandem mass tag
WES: Whole-exome sequencing
