# Supplementary figures and images for: Genotyping, generation and proteomic profiling of the first human autosomal dominant osteopetrosis type II-specific induced pluripotent stem cells
Source: Stem Cell Res Ther. 2019 Aug 14;10:251. doi: 10.1186/s13287-019-1369-8 (PMC6693165; doi:10.1186/s13287-019-1369-8)

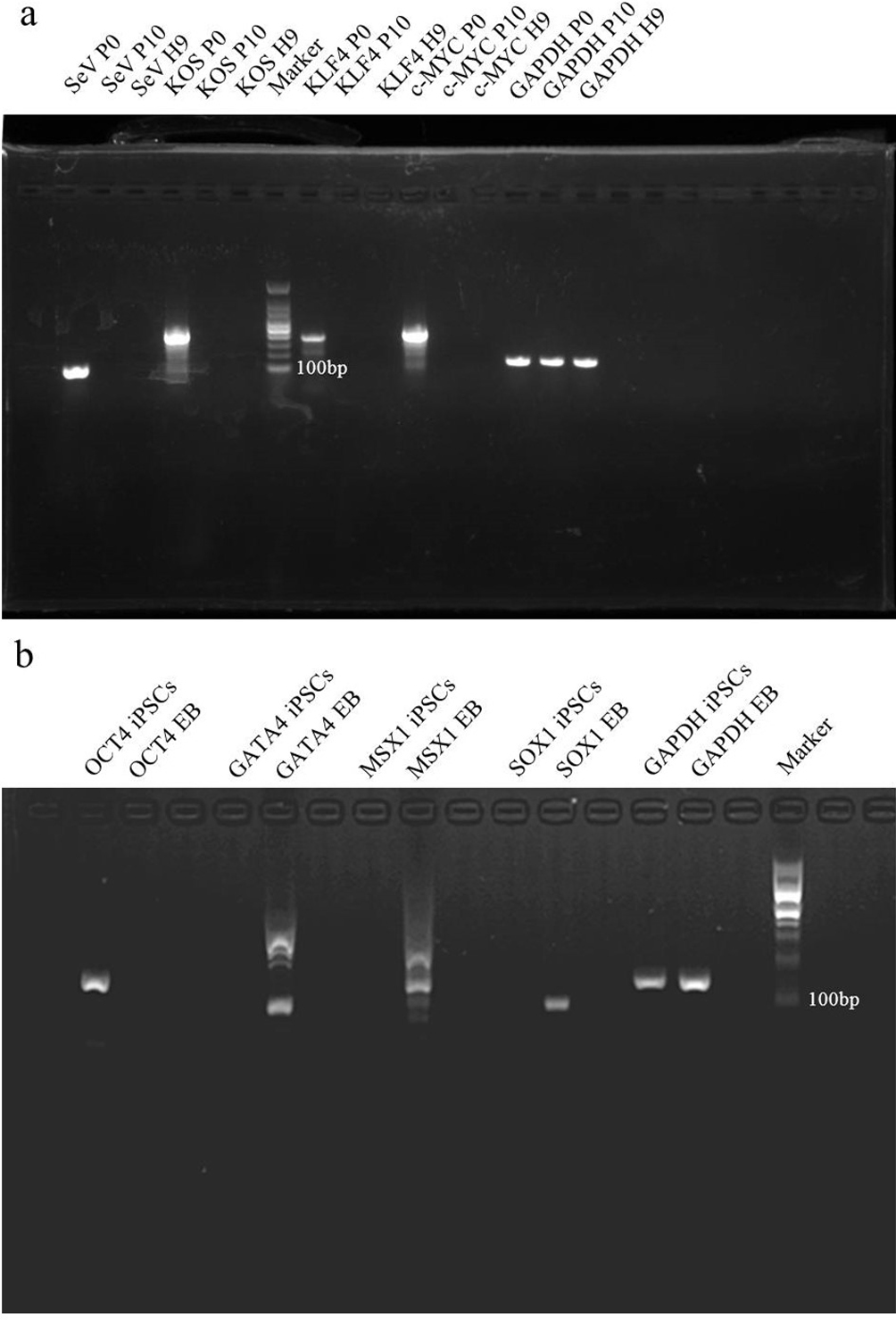

Supplement: Supplementary file 1 — Figure S1. Uncropped and full-length gels. The displayed gels correspond to the following figures of the main text: (a) Fig. 4a. (b) Fig. 4b. (JPG 337 kb) [file 13287_2019_1369_MOESM1_ESM.jpg]

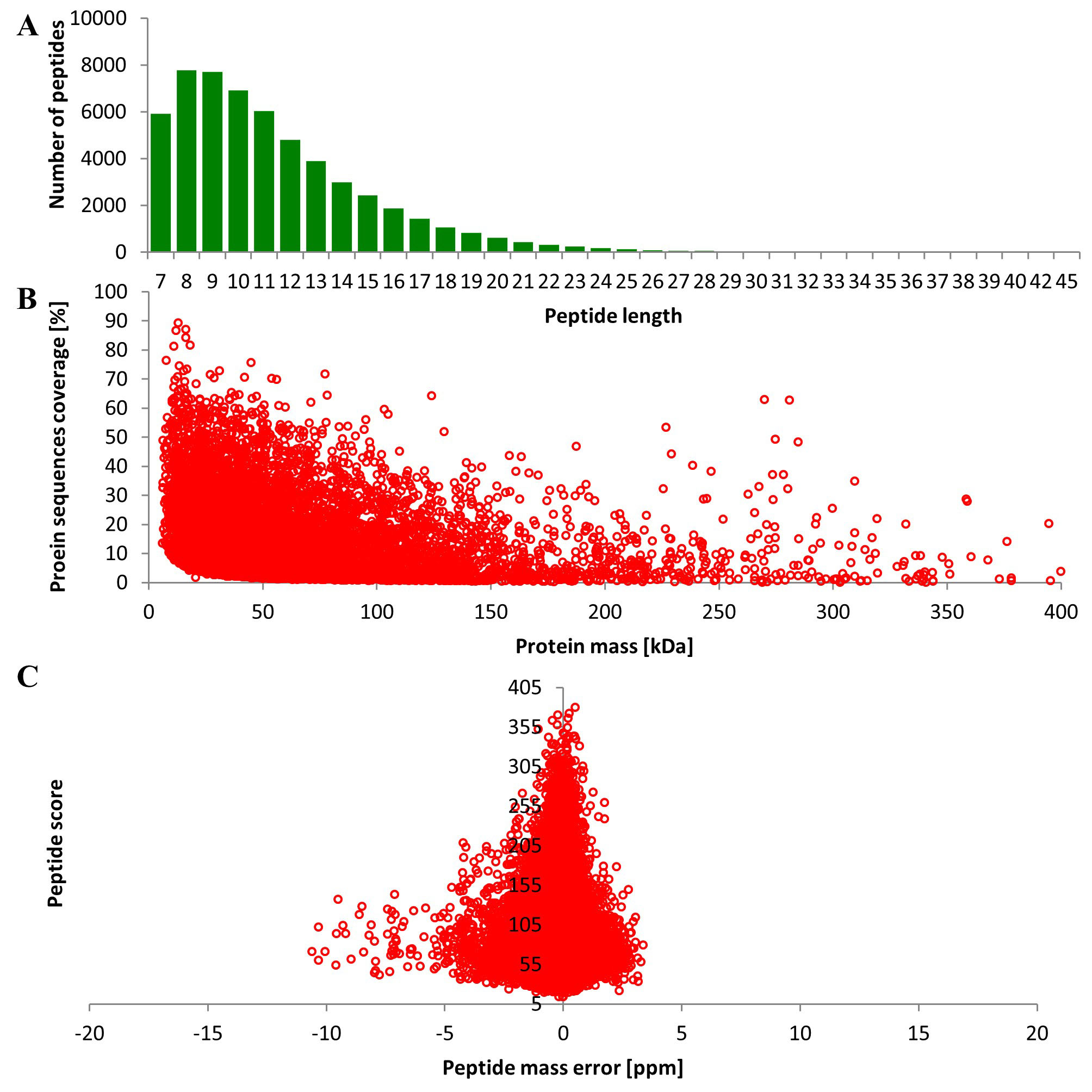

Supplement: Supplementary file 2 — Figure S2. Quality control of the MS data. (A). The length distribution of the identified peptide. (B). Relationship between the identified protein mass and coverage. (C). Mass precision distribution of MS data. (JPG 438 kb) [file 13287_2019_1369_MOESM2_ESM.jpg]

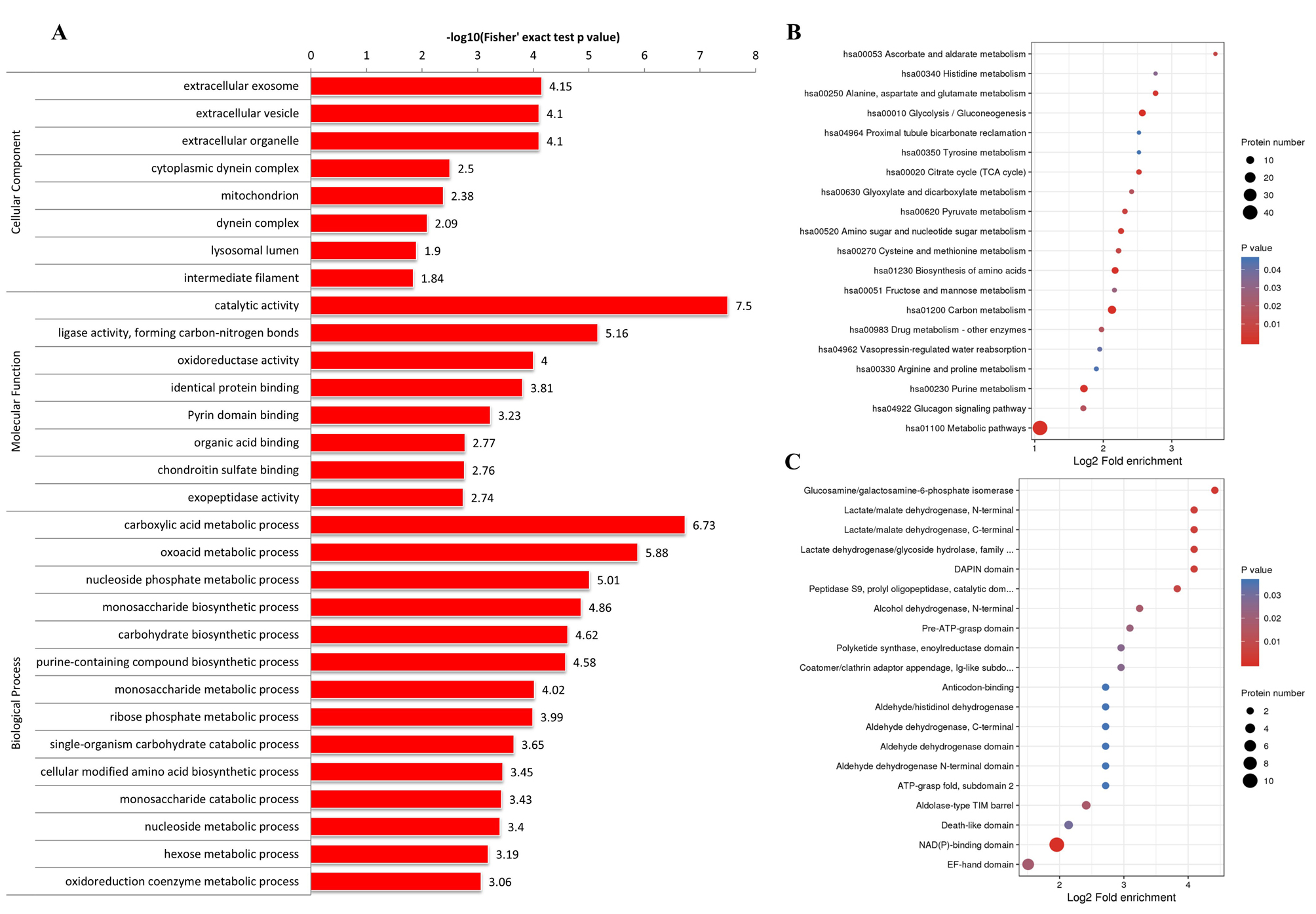

Supplement: Supplementary file 3 — Figure S3. Functional enrichment analysis of the DEPs in the ADO2-iPSCs. (A). GO-based functional enrichment analysis. (B). KEGG-based functional enrichment analysis. (C). Protein domain enrichment analysis. (JPG 833 kb) [file 13287_2019_1369_MOESM3_ESM.jpg]

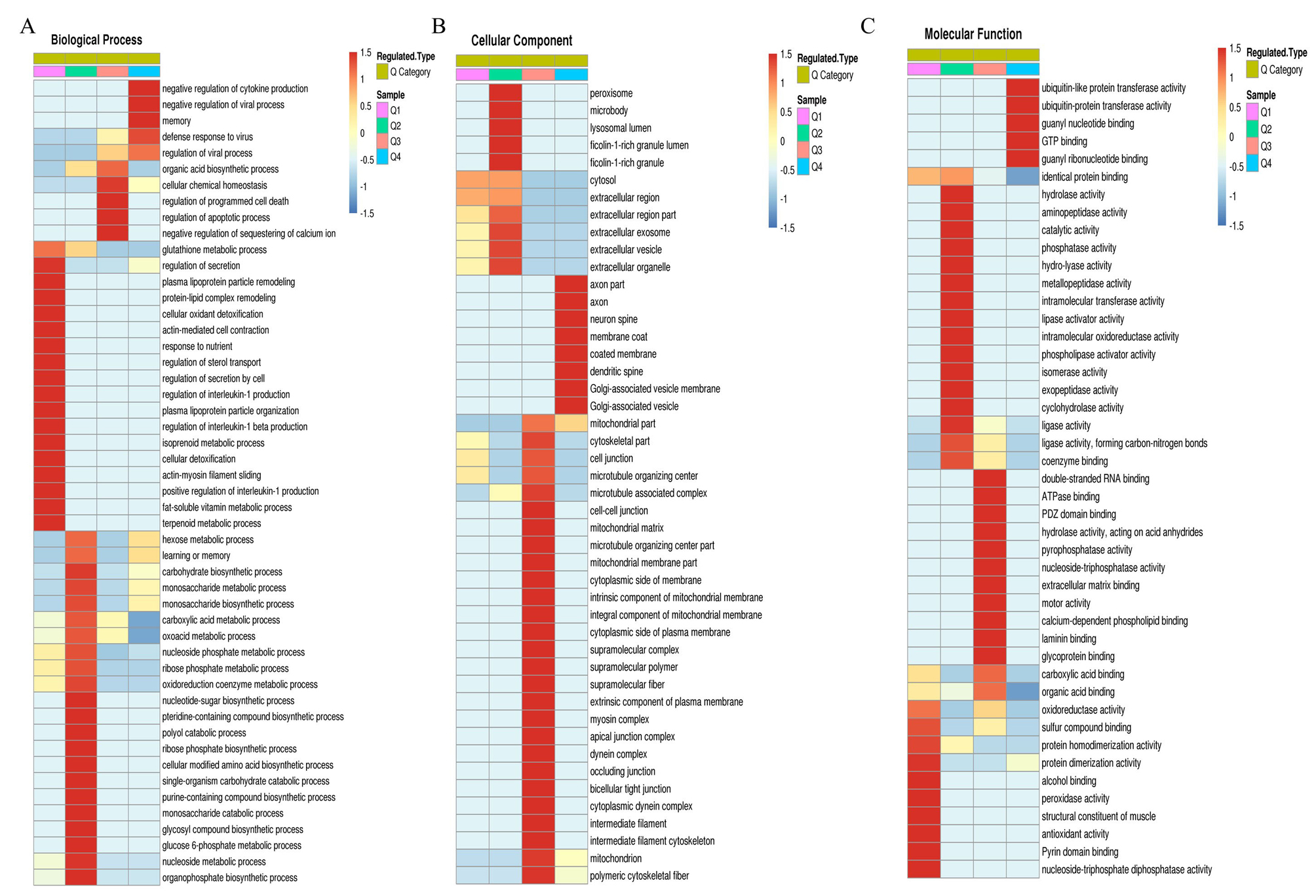

Supplement: Supplementary file 4 — Figure S4. GO functional enrichment clustering analysis of the DEPs of ADO2-iPSCs. All of the DEPs were divided into four quantiles (Q1–Q4) according to fold changes: Q1 (0 < ratio < 0.77), Q2 (0.77 < ratio < 0.83), Q3 (1.2 < ratio < 1.3), and Q4 (ratio > 1.3), and further performed GO, KEGG pathway and protein domain functional enrichment clustering analysis. (A). Biological process. (B). Cellular component. (C). Molecular function. (JPG 1015 kb) [file 13287_2019_1369_MOESM4_ESM.jpg]

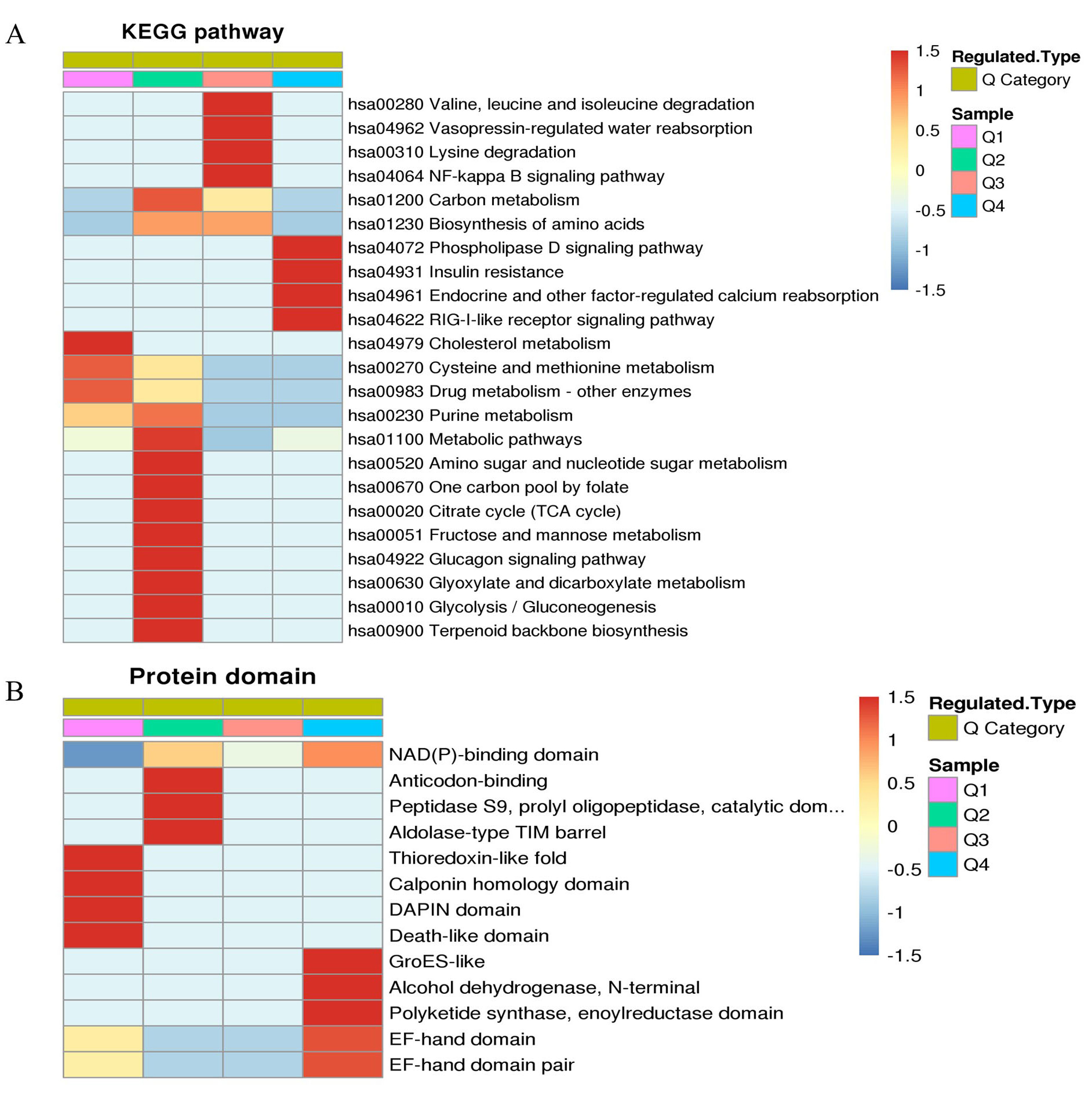

Supplement: Supplementary file 5 — Figure S5. KEGG pathway (A) and protein domain (B) functional enrichment clustering analysis of the DEPs in ADO2-iPSCs. (JPG 1117 kb) [file 13287_2019_1369_MOESM5_ESM.jpg]

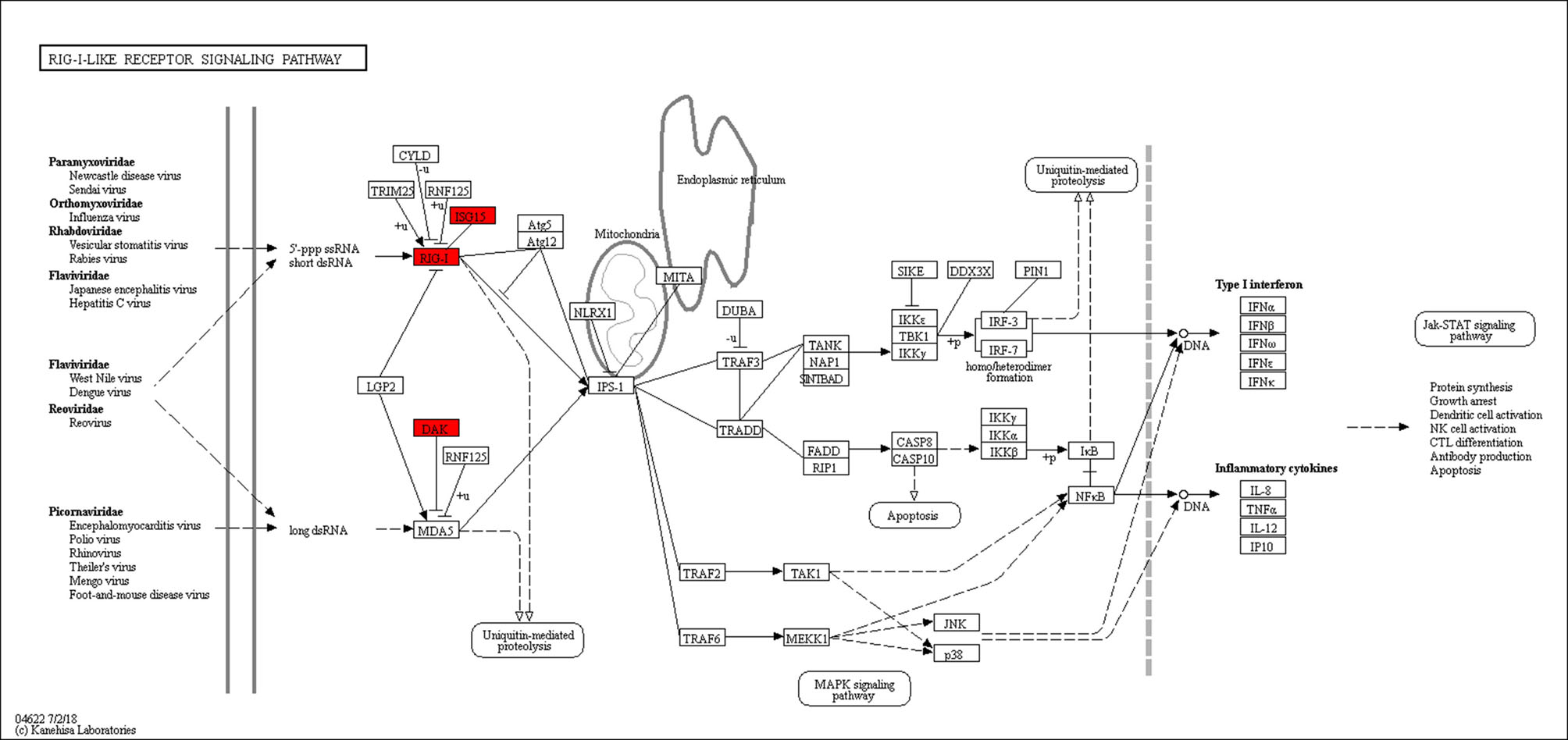

Supplement: Supplementary file 6 — Figure S6. The KEGG RIG-I-like receptor signaling pathway (red represents upregulated, green represents downregulated, yellow indicates that there are multiple proteins in this node, including differentially upregulated and downregulated proteins.). (JPG 200 kb) [file 13287_2019_1369_MOESM6_ESM.jpg]

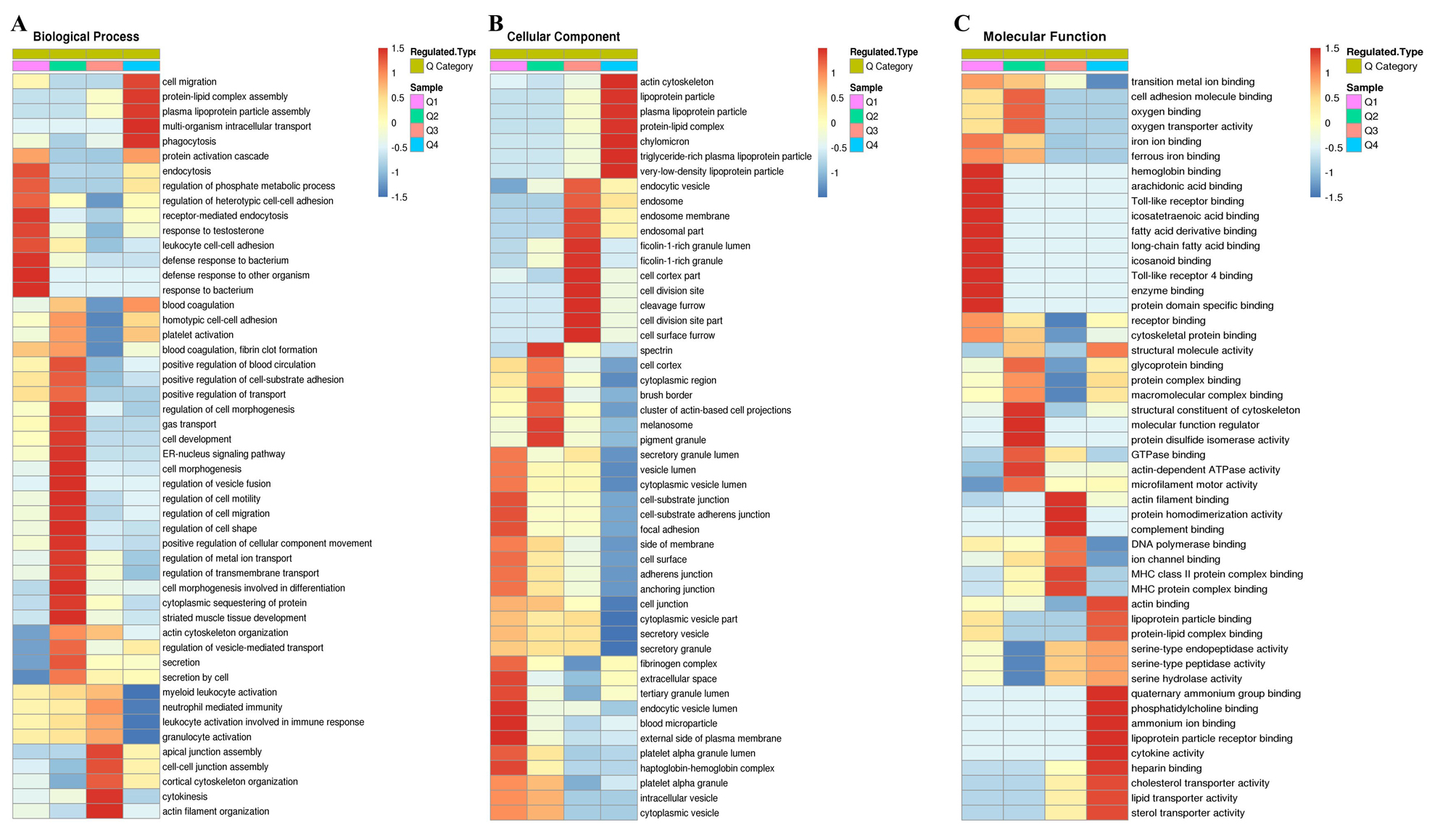

Supplement: Supplementary file 7 — Figure S7. GO functional enrichment clustering analysis of the differently Khib-modified proteins in ADO2-iPSCs. All of the differently Khib-modified proteins were divided into four quantiles (Q1–Q4) according to fold changes: Q1 (0 < ratio < 0.77), Q2 (0.77 < ratio < 0.83), Q3 (1.2 < ratio < 1.3), and Q4 (ratio > 1.3), and further performed GO, KEGG pathway and protein domain functional enrichment clustering analysis. (A). Biological process. (B). Cellular component. (C). Molecular function. (JPG 930 kb) [file 13287_2019_1369_MOESM7_ESM.jpg]

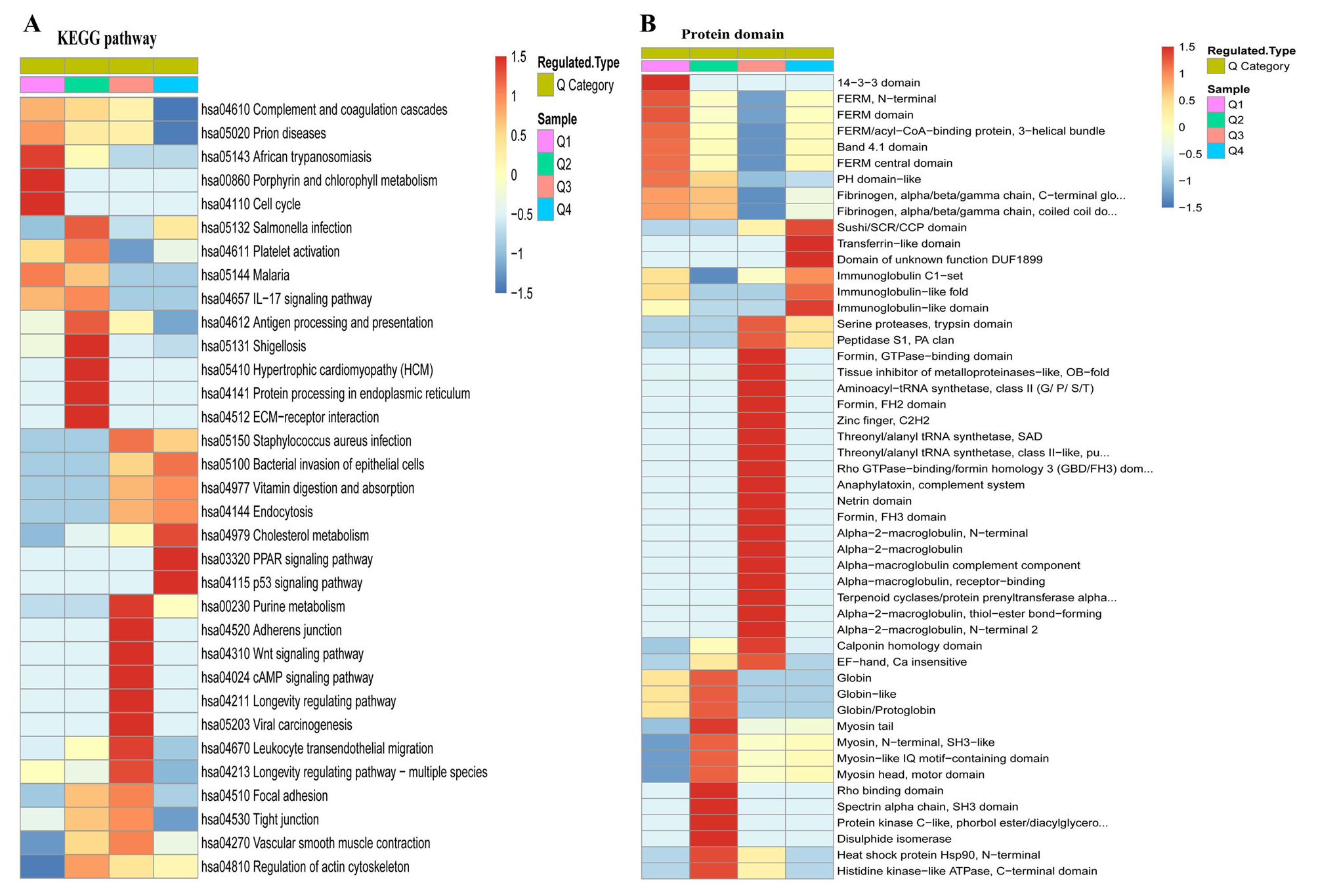

Supplement: Supplementary file 8 — Figure S8. KEGG pathway (A) and Protein domain (B) functional enrichment clustering analysis of the differently Khib-modified proteins in the ADO2-iPSCs. (JPG 963 kb) [file 13287_2019_1369_MOESM8_ESM.jpg]
