# Supplementary material for: Genotyping, generation and proteomic profiling of the first human autosomal dominant osteopetrosis type II-specific induced pluripotent stem cells
Source: Stem Cell Res Ther. 2019 Aug 14;10:251. doi: 10.1186/s13287-019-1369-8 (PMC6693165; doi:10.1186/s13287-019-1369-8)
Supplement: Supplementary file 10 — Full proteomic analysis methods. (DOCX 21 kb) [file 13287_2019_1369_MOESM10_ESM.docx]

**Full proteomic analysis methods**

**Cell samples and Protein Extractions**

To characterize the ADO2-iPSCs by proteomics, peptides were preparared using the ADO2-iPSCs and normal control iPSCs (NC-iPSCs) that were induced from the urine of a healthy human donor and provided by Cellapy Biotechnology (Beijing, China). The NC-iPSCs was consider as a standard iPSC line with well-known characteristics, and our ADO2-iPSCs was generated using the same way. Cells were preprocessed in lysis buffer (8 M urea, 1% Protease Inhibitor Cocktail) and sonicated three times on ice using a high intensity ultrasonic processor (Scientz Biotechnology, Ningbo, China). Afer centrifugation at 12,000 g at 4 °C for 10 min, the remaining precipitate were removed, and collected the supernatant. And then, we determined the protein concentration with BCA Kit according to the manufacturer’s recommended methods. The protein extractions were stored at -80 °C for further proteomic experiments. The present study was performed in accordance with the Helsinki Declaration and approved by the Ethics Committee of Shenzhen People's Hospital and the Guilin No. 181 Hospital.

**Trypsin digestion**

For digestion, the protein solution was processed with 5 mM dithiothreitol ( Sigma ) for 30 min at 56 °C and alkylated with 11 mM iodoacetamide (Sigma) for 15 min at room temperature in darkness. To eliminate the efects of urea in trypsin digestion, 100 mM TEAB was added to the protein sample, and diluted urea concentration in trypsin digestion less than 2M. The trypsin (Promega) was added at 1:50 trypsin-to-protein mass ratio for the first digestion overnight and 1:100 trypsin-to-protein mass ratio for a second 4 h-digestion.

**TMT Labeling**

After trypsin digestion, peptides were desalted by Strata X C18 SPE column (Phenomenex, Torrance, CA) and vacuum-dried. Peptides were reconstituted in 0.5 M TEAB and processed according to the manufacturer’s protocol for 6-plex TMT Kit. Briefly, one unit of TMT Reagent (Thermo, defined as the amount of reagent required to label 1 mg of protein) were thawed and reconstituted in acetonitrile. The peptide mixtures were then incubated for 2 h at room temperature and pooled, desalted and dried by vacuum centrifugation.

**HPLC Fractionation**

The tryptic peptides were fractionated into fractions by high pH reverse-phase HPLC using Agilent 300Extend C18 column (5 μm particles, 4.6 mm ID, 250 mm length). Briefly, peptides were first separated with a gradient of 8% to 32% acetonitrile (pH 9.0) over 60 min into 60 fractions. Then, the peptides were combined into 1 fractions and dried by vacuum centrifuging.

**Affinity enrichment of lysine K_hib_-modified peptides**

Lysine K_hib_-modified peptides were enriched by immunoprecipitation. Briefly, the

fractionated peptides were dissolved in NETN buffer (100 mM NaCl, 1 mM EDTA, 50 mM Tris-HCl, 0.5% NP-40, pH 8.0) were incubated with pre-washed antibody beads (Lot number: PTM804, PTM Bio, Hangzhou, China) at 4°C overnight with gentle shaking. The beads were subsequently washed with NETN buffer four times and twice with H_2_O. The bound peptides were eluted from the beads with 0.1% trifluoroacetic acid. Finally, the eluted fractions were combined and vacuum-dried. For LC-MS/MS analysis, the resulting peptides were desalted with C18 ZipTips (Millipore) according to the manufacturer’s instructions.

**LC-MS/MS Analysis**

The tryptic peptides were dissolved in 0.1% formic acid (solvent A), directly loaded onto a home-made reversed-phase analytical column (15-cm length, 75 μm i.d.). The gradient was comprised of an increase from 6% to 23% solvent B (0.1% formic acid in 98% acetonitrile) over 26 min, 23% to 35% in 8 min and climbing to 80% in 3 min then holding at 80% for the last 3 min, all at a constant flow rate of 400 nL/min on an EASY -nLC 1000 UPLC system.

The peptides were subjected to NSI source followed by tandem mass spectrometry (MS/MS) in Q Exactive^TM^ Plus (Thermo) coupled online to the UPLC. The electrospray voltage applied was 2.0 kV. The m/z scan range was 350 to 1800 for full scan, and intact peptides were detected in the Orbitrap at a resolution of 70,000. Peptides were then selected for MS/MS using NCE setting as 28 and the fragments were detected in the Orbitrap at a resolution of 17,500. A data-dependent procedure that alternated between one MS scan followed by 20 MS/MS scans with 15.0s dynamic exclusion. Automatic gain control (AGC) was set at 5E4. Fixed first mass was set as 100 m/z.

**Database Search**

Maxquant search engine (v.1.5.2.8) was used to process the resulting MS/MS data. Tandem MS were searched against the SwissProt Human database concatenated with reverse decoy database. Trypsin/P was specified as cleavage enzyme allowing up to 5 modifcations, 4 missed cleavages per peptide and max. 7 charges.. The mass tolerance for precursor ions was set as 20 ppm in First search and 5 ppm in Main search, and the mass tolerance for fragment ions was set as 0.02 Da. Carbamidomethyl on Cys was specified as fixed modification and K_hib_ modification and oxidation on Met were specified as variable modifications. FDR was adjusted to < 1% and minimum score for modified peptides was set > 40.

**Bioinformatics analysis**

***Motif and Subcellular Localization Analysis***

MoMoV5.0.2 was used to analysis the model of sequences constituted with amino acids in specific positions of modify-21-mers (10 amino acids upstream and downstream of the site, but phosphorylation with modify-13-mers that 6 amino acids upstream and downstream of the site) in all protein sequences. And all the database protein sequences were used as background database parameter. Minimum number of occurrences was set to 20. Emulate original motif-x was ticked, and other parameters with default. Subsequently, Wolfpsort v.0.2 was selected to perform the subcellular localization analysis.

***Functional enrichment*** ***and clustering analysis***

Gene Ontology (GO) annotation was performed using the UniProt-GOA database (www. http://www.ebi.ac.uk/GOA/). Firstly, Converting identified protein ID to UniProt ID and then mapping to GO IDs by protein ID. If some identified proteins were not annotated by UniProt-GOA database, the InterProScan v.5.14-53.0 (http://www.ebi.ac.uk/interpro/) would be used to annotated protein’s GO functional based on protein sequence alignment method. The K_hib_-modifed proteins were then further classified by GO annotation based on three categories: biological process, cellular component and molecular function.

The Kyoto Encyclopedia of Genes and Genomes (KEGG) database was used to annotate protein pathway. Firstly, using KEGG online service tools KAAS v.2.0 (http://www.genome.jp/kaas-bin/kaas_main) to annotated protein’s KEGG database description. Then mapping the annotation result on the KEGG pathway database using KEGG online service tools KEGG mapper V2.5 (http://www.kegg.jp/kegg/mapper.html). Identified 2-hydroxyisobutyrylated proteins domain functional description were annotated by InterProScan on InterPro domain database. All of the bioinformatics analysis, including GO, KEGG pathway and protein domain enrichment analysis, were performed by a two-tailed Fisher’s exact test. Each GO, KEGG pathway and protein domain term with a corrected p-value < 0.05 is considered significant.

Then, we divided the differentially K_hib_-modifed proteins into four quantiles (Q1−Q4) according to fold changes: Q1 (0 < ratio < 0.77), Q2 (0.77 < ratio < 0.83), Q3 (1.2 < ratio <1.3), and Q4 (ratio >1.3), and further performed functional enrichment clustering analysis. Firstly, we collated all the categories that were obtained after enrichment along with their P values, and then filtered for those categories which were at least enriched in one of the clusters with P value <0.05. This filtered P value matrix was transformed by the function x = −log10 (P value). Finally these x values were z-transformed for each functional category. These z scores were then clustered by one-way hierarchical clustering (Euclidean distance, average linkage clustering) in Genesis. Cluster membership were visualized by a heat map using the “heatmap.2” function from the “gplots” R-package v.2.0.3 (<https://cran.r-project.org/web/packages/cluster/>).

***The potential relationship analysis*** ***between DEPs, K_hib_-modifed proteins and ADO2***

In order to search the potential relationship between DEPs, K_hib_-modifed proteins and ADO2, we mapped the DEPs, the K_hib_-modifed proteins and three target genes (MITF, TFE3 and CLCN7) by the STRING website (<https://string-db.org/>). STRING defines a metric called the confidence score to define the confidence of the interaction. And then, we fetched all interactions that had a confidence score ≥ 0.9, and our interaction network form STRING was visualized by Cytoscape 3.6.1.
